# In Situ Polymerization in COF Boosts Li-Ion Conduction in Solid Polymer Electrolytes for Li Metal Batteries

**DOI:** 10.1007/s40820-025-01768-3

**Published:** 2025-05-06

**Authors:** Junchen Meng, Mengjia Yin, Kairui Guo, Xingping Zhou, Zhigang Xue

**Affiliations:** Key Laboratory of Material Chemistry for Energy Conversion and Storage, Ministry of Education, Hubei Key Laboratory of Material Chemistry and Service Failure, School of Chemistry and Chemical Engineering, Huazhong University of Science and Technology, Wuhan, 430074 People’s Republic of China

**Keywords:** Covalent organic framework, In situ polymerization, Ring-opening polymerization, Solid polymer electrolyte, Lithium metal batteries

## Abstract

Solid polymer electrolytes (SPEs) have garnered considerable interest in the field of lithium metal batteries (LMBs) owing to their exceptional mechanical strength, excellent designability, and heightened safety characteristics. However, their inherently low ion transport efficiency poses a major challenge for their application in LMBs. To address this issue, covalent organic framework (COF) with their ordered ion transport channels, chemical stability, large specific surface area, and designable multifunctional sites has shown promising potential to enhance lithium-ion conduction. Here, we prepared an anionic COF, TpPa-COOLi, which can catalyze the ring-opening copolymerization of cyclic lactone monomers for the in situ fabrication of SPEs. The design leverages the high specific surface area of COF to facilitate the absorption of polymerization precursor and catalyze the polymerization within the pores, forming additional COF-polymer junctions that enhance ion transport pathways. The partial exfoliation of COF achieved through these junctions improved its dispersion within the polymer matrix, preserving ion transport channels and facilitating ion transport across COF grain boundaries. By controlling variables to alter the crystallinity of TpPa-COOLi and the presence of –COOLi substituents, TpPa-COOLi with partial long-range order and –COOLi substituents exhibited superior electrochemical performance. This research demonstrates the potential in constructing high-performance SPEs for LMBs.

**Supplementary Information:**

The online version contains supplementary material available at 10.1007/s40820-025-01768-3.

## Highlights

- Solid polymer electrolytes formed in situ via covalent organic framework-induced ring-opening copolymerization.
- Solid polymer electrolytes with a high lithium-ion transference number and desirable interfacial compatibility.
- Li⁺ migration mechanisms investigated with density functional theory and molecular dynamics simulations.

**Supplementary Information:**

The online version contains supplementary material available at 10.1007/s40820-025-01768-3.

## Graphical abstract

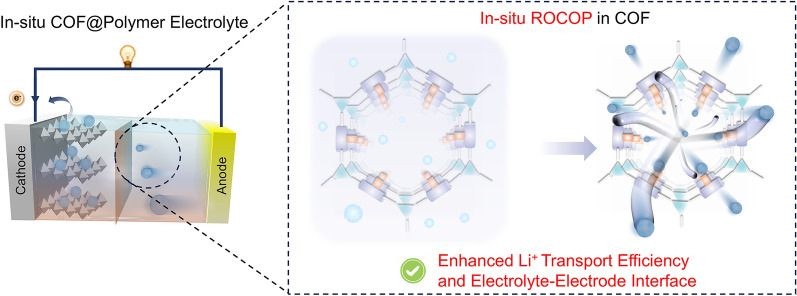

## Introduction

As superior ion carriers, solid polymer electrolytes (SPEs) typically necessitate good lithium salt solubility, high lithium-ion transport efficiency, low electrode–electrolyte interface impedance, and an electrochemical stability window that is compatible with the positive electrode material [1, 2]. Polyester-based SPEs are extensively studied due to their stable electrochemical performance and higher lithium-ion transference numbers compared to polyether-based polymer electrolytes [3–6]. The consensus within the researchers is the primary challenge that constrains the utilization of SPEs in all-solid-state batteries stemming from their inherently low ambient ionic conductivity (< 10^–7^ S cm^−1^) and low lithium-ion transference number (< 0.5) in room temperature [7, 8]. Incorporating functional fillers within polymer electrolyte as a compensatory agent is a charming modification way to improve the low ion transport efficiency of SPEs [9]. The conventional ex situ composite method, which involves dissolving the polymer with a suitable solvent, dispersing the fillers in the polymer solution, and subsequently fabricating solid electrolyte membranes through pouring, hot pressing, coating, or other techniques, often fails to address the issue of uneven filler dispersion and extremely high interface impedance [10, 11]. The reason is that the inherent defects of the fillers, such as high zeta potential and low specific surface area, can lead to aggregation and reduced utilization rates, particularly at high fillers concentrations, resulting in poor tensile strength of the electrolyte membrane, sluggish ion transport kinetics, and uneven ion flux [8, 12]. Furthermore, the ex situ assembly battery method employed in the preparation of solid-state electrolyte membranes still exhibits high interface impedance between the electrode, which hinders their application in solid-state batteries [13, 14].

Long-range ordered porous materials, such as covalent organic frameworks (COFs) [15], metal–organic frameworks (MOFs) [16–18], and zeolites [19], have been widely used in energy storage materials owning to their unique channels for orderly ion transport, good chemical structural stability, large specific surface area, and strong designability of multifunctional sites. COFs are a class of porous crystalline polymer materials assembled into 2D or 3D long-range ordered periodic structures through highly designable building blocks of organic monomers linked by covalent bonds [20, 21]. The stable chemical structure and high modulus of COFs ensure good dendrite suppression, and their defined nanoscale channels endow them with high ambient ionic conductivity (> 10^–5^ S cm^−1^) and lithium-ion transference number (> 0.6) at room temperature [20–25]. However, due to the inherent disadvantage of powder materials as solid electrolytes, lithium ions are difficult to conduct between grain boundaries in COFs, and there is a large interface impedance at the interface contact with the electrode which restricts its performance during long battery cycles as a “shortboard effect” [26–30]. A promising research direction entails the employment of COFs and polymer composite electrolyte, capitalizing on the advantageous soft interfacial contact of polymers to substitute the high-contact-impedance solid–solid interfaces of powder materials and assistant the ion transport in the grain boundary of COFs [31–35]. This blending composite can also effectively enhance the ion transport efficiency of polymers theoretically as multifunctional fillers. The methodology seems feasible due to the capacity of polymers to utilize in situ polymerization techniques to alleviate their intrinsic high interfacial impedance challenges. Notably, COFs are difficult to stably disperse in the organic solvent especially the precursor solution of monomers and Li salts, which cause the deposition of COFs on the side where the electrolyte precursor solution is dropped. Hence, there has been limited utilization of in situ polymerization technology in relevant research endeavors pertaining to COF-based SPEs. According to previous work reports [34], when polymers are ex situ blended with COFs, the interaction between the polymer and COFs is primarily a surface interaction, and it is difficult for the polymer to penetrate deeply into the pores of COFs, thus unable to effectively utilize the advantages of COFs in ion conduction and assist in ion conduction at the inner walls and grain boundaries of the COFs. Therefore, the key to achieving SPEs with efficient fast-ion transport lies in effectively enhancing the utilization rate of COFs pores to ensure COFs uniform dispersion within polymers, while concurrently addressing the challenge of high electrode–electrolyte interface impedance that SPEs inherently possess.

**Fig. 1:**
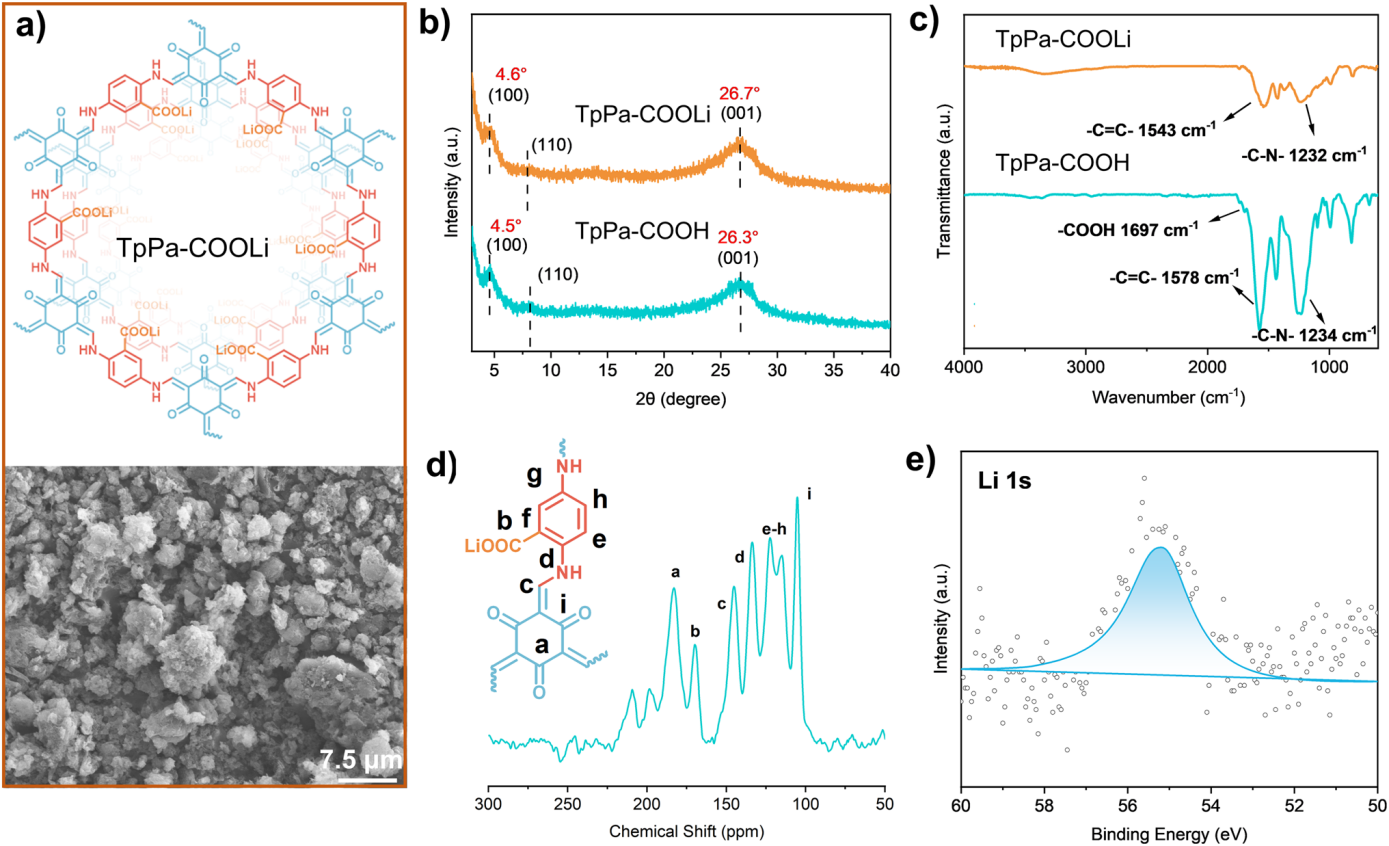
**a** Chemical structure and SEM image of TpPa-COOLi. **b** PXRD patterns and **c** FTIR spectra of TpPa-COOH and TpPa-COOLi. **d** Solid-State ^13^C NMR spectrum of TpPa-COOLi. **e** XPS (Li 1*s*) spectrum of TpPa-COOLi

Herein, an anionic COF, TpPa-COOLi (Fig. 1a), which is capable of catalyzing the ring-opening copolymerization (ROCOP) of cyclic lactone monomers of ε-caprolactone (CL) and trimethylene carbonate (TMC), was prepared and can be utilized for the in situ fabrication of SPEs. The in situ design capitalizes on the high specific surface area of COF to facilitate the absorption of polymerization precursor liquids and to catalyze ROCOP of CL and TMC within the pores via the -COOLi single-ion sites located on the COF, leading to the formation of additional COF-polymer junctions to construct fast-ion transport pathway. Significantly, the partial exfoliation of COF was achieved by the creation of numerous COF-polymer junctions, which in turn enhanced the dispersion of COF within the polymer matrix, and this process preserved the majority of ion transport channels. Moreover, the ROCOP within the COF pores facilitated ion transport across the COF grain boundaries, and monomers situated outside the pores underwent in situ polymerization, resulting in a reduction of the interfacial impedance of the electrode–electrolyte interface. In this study, different SPEs were prepared by controlling variables to alter the crystallinity of TpPa-COOLi in the presence or absence of –COOLi substituents. Electrochemical testing results showed that TpPa-COOLi with partial long-range order and –COOLi substituents on the COF exhibited superior electrochemical performance, which strongly shows the potential of in situ polymerization in COF for constructing SPEs.

## Results and Discussion

### Characterizations of COF and COF-Based Separator

TpPa-COOH was synthesized through a Schiff base condensation reaction using 1,3,5-tri(4-formylphenyl)benzene and 2,5-diaminobenzoic acid. Then, the cation exchange was performed from H^+^ to Li^+^ using Li_2_CO_3_ to form TpPa-COOLi according to the reported method (Fig. S1) [26]. Powder X-ray diffraction (PXRD) measurements showed strong characteristic peaks at 4.5° and 26.3°, corresponding to 2D layered structures and interlayer π-π stacking interactions (Fig. 1b), consistent with previously reported characteristic peak results. The characteristic Fourier transform infrared (FTIR) spectrum confirmed the formation of characteristic ketone enamine bonds at 1230 cm^−1^ in TpPa-COOLi (Fig. 1c). In addition, solid-state ^13^C NMR further demonstrates the structural uniformity (Fig. 1d). X-ray photoelectron spectroscopy (XPS) spectrum of Li 1*s* (Fig. 1e) of TpPa-COOLi shows that a new Li peak exists at 55.25 eV. The observed upshift in the binding energies of the C 1*s* and O 1*s* peaks in the XPS spectra (Figs. S2 and S3) post-lithiation originates from the ionic coordination between carboxyl oxygen atoms and Li⁺. This interaction induces electron density redistribution toward the oxygen centers through Li⁺-induced polarization, thereby increasing the effective electronegativity of the oxygen atoms. Consequently, the enhanced electron density localization at the oxygen sites leads to an upshift in the binding energies of the corresponding XPS peaks, as evidenced by the characteristic spectral shifts in Fig. S3. These spectroscopic signatures collectively confirm successful lithium intercalation within the TpPa-COOH framework [26].

**Fig. 2:**
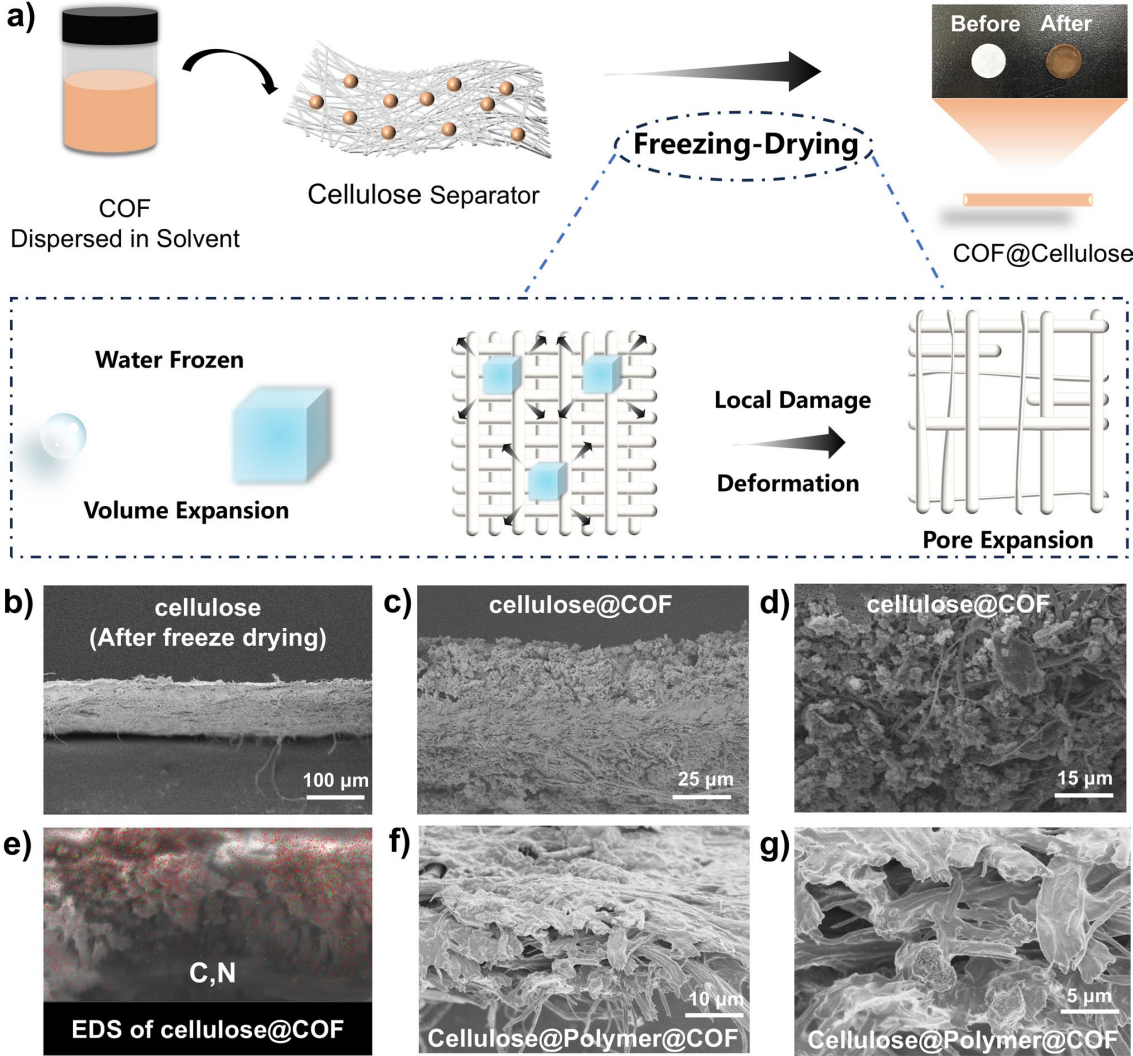
**a** Process of loading COF onto expanded pore cellulose separators through freeze-drying. **b** SEM section view image of cellulose separator after freeze-drying treatment. **c, d** SEM section view image of cellulose separator after freeze-drying treatment. **e** EDS characterization of the section view image of cellulose separator loaded COF. **f, g** Local magnification SEM section view image of cellulose separator loaded COF and polymer

The COF was impregnated onto the cellulose separators via a freeze-drying method (Fig. 2a), which is beneficial to enhancing compatibility with the in situ polymerization technology, with the aim of addressing the issue of elevated interfacial impedance associated with COF-based electrolytes. The particle size of COF powder, finely ground through mechanical ball milling, remains at the micrometer level (Fig. 1a), which is not significantly distinct from the pore size of the cellulose separators, rendering it challenging for COF to permeate the entire interior and both surfaces of the cellulose separators. Commercial cellulose separators possess dense and uniform pore sizes, further impeding the entry of COF particles into the internal pores of separators. In the process of freeze-drying to expand the pore size of the cellulose separator (Fig. 2a), as water freezes into ice, it undergoes volume expansion, and the expansion of ice crystals exerts pressure on the pore walls of the separator, causing deformation or local damage, thereby facilitating the enlargement of the internal pores of the separator. When the ice crystals contained within the internal pores of the separator freeze and sublime, the space originally occupied by the ice crystals becomes vacant, forming larger pores or channels. Consequently, the pores of the cellulose separator treated by this process become loose and swollen, and the thickness of the separator also expands (Figs. 2b and S6).

Detailed scanning electron microscopy (SEM) was employed to characterize the cross section of the cellulose separator after freeze-drying treatment and loaded with COF (Fig. 2c, d). The results indicate that COF was successfully loaded onto both sides and within the cellulose separator. The energy-dispersive spectroscopy (EDS) results reveal that COF is relatively uniformly dispersed within the pores of the cellulose separator (Figs. 2e and S7). To observe the composite of the polymer, cellulose separators, and COF inside the battery, a cellulose separator loaded with TpPa-COOLi was assembled to a battery where polymer and TpPa-COOLi were in situ blended. The battery was then disassembled without any pretreatment to remove the cellulose separator. SEM was used to observe its cross-sectional morphology (Fig. 2f, g), which demonstrated that the polymer tightly filled the pores of the cellulose separators, forming a dense structure and wrapping around the fibers. The gaps between COF particles were also filled with the polymer matrix, achieving the objective of an in situ composite of COF and polymer.

We further prepared dog-bone-shaped specimens of these cellulose separators and loaded them with polymers and COF to elucidate the substantial impact of these components on their mechanical properties (Fig. S8). The results show that the original cellulose separator has poor mechanical properties, with a maximum tensile stress of only 4.5 MPa and a tensile strain of just 2.2%. When loaded with a polymer (Cellulose@Polymer), the mechanical strength decreases further. This is because the polymer disrupts the hydrogen bonds in the cellulose separator. Specifically, the maximum tensile strength drops significantly to 1.9 MPa, while the tensile strain increases threefold to 6.6%. After the separator is loaded with a COF, its mechanical properties improve markedly, with increased modulus, maximum tensile stress rising to 10.3 MPa, and a less significant strain change of only 3.6%. This significant enhancement in the separator's modulus and mechanical properties effectively suppresses lithium dendrite growth. Traditional polymer electrolytes, often made from soft polyester-based materials to ensure ion conductivity, lack sufficient mechanical strength. In contrast, the COF-loaded separator greatly boosts the overall mechanical properties of SPEs. Although the maximum tensile strength of cellulose@polymer@COF decreases to 5.7 MPa, it remains higher than that of the original cellulose separator and offers a more flexible tensile strain, which helps accommodate the volume expansion during lithium dendrite growth.

### Characterizations of Copolymer and Polymer Electrolytes

**Fig. 3:**
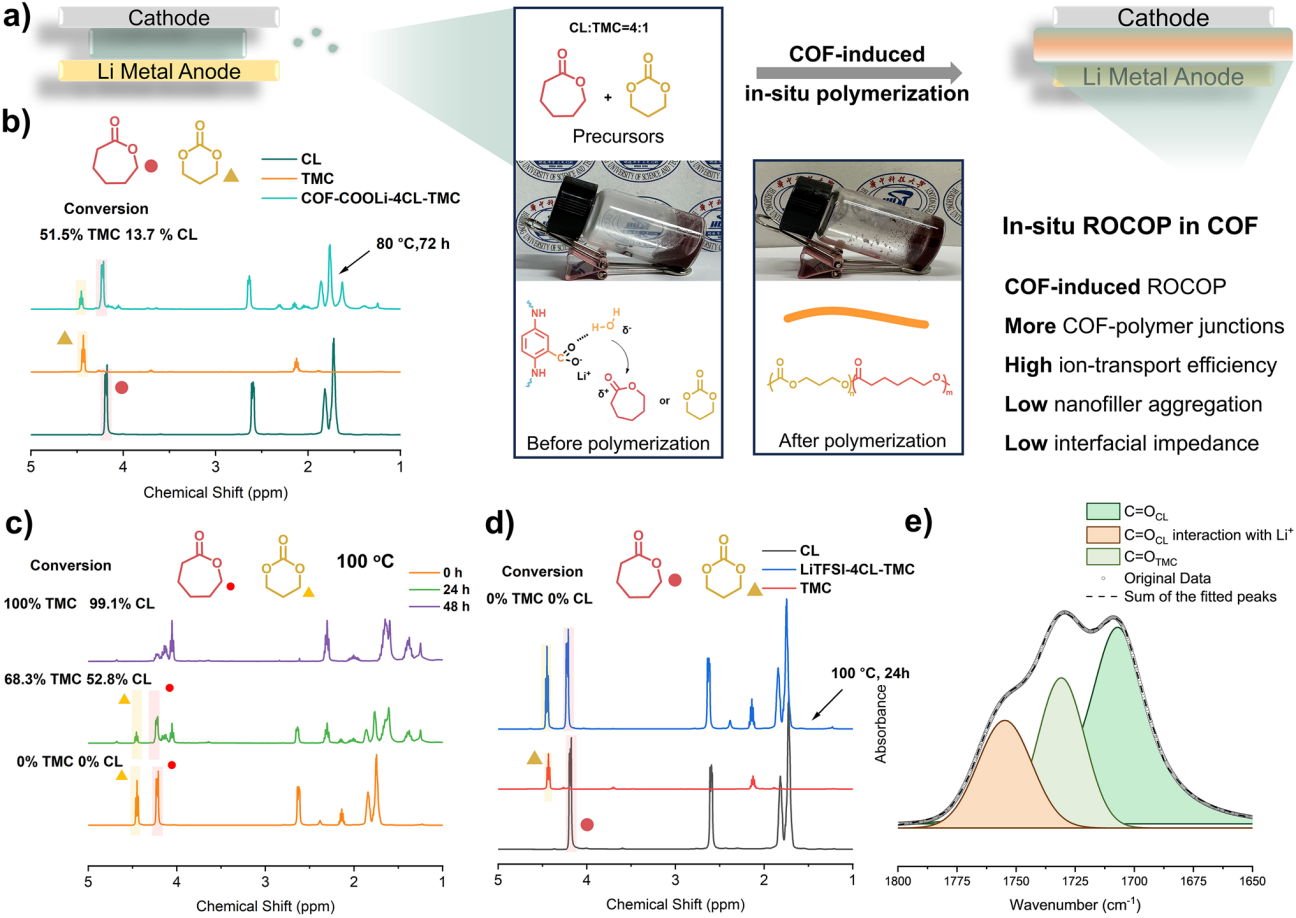
**a** Digital images of polymer@TpPa-COOLi before polymerization and after polymerization. ^1^H NMR spectra (CDCl_3_) of P(4CL-TMC) catalyzed by TpPa-COOLi at **b** 80 °C and **c** 100 °C. **d** ^1^H NMR spectra (CDCl_3_) of 4CL-TMC monomer precursor solution with 30 wt% LiTFSI. **e** FTIR spectra of carbonyl groups at 1800–1650 cm^−1^ in 4CL-TMC monomer precursor solution with 30 wt% LiTFSI

The optimal ratio of CL-TMC copolymerization (molar ratio 4:1) was used in a reported work to optimize electrochemical performances of SPEs [36]. The ring-opening polymerization of cyclic lactones generally requires the catalysis of a small amount of proton acid [37], Lewis acid [38], Lewis base [39], or Lewis base with urea [40]. Drawing upon the chemical structure of these catalysts, the catalysis of lactone monomers via carboxylate salts appears to be a relatively straightforward approach to implement on the pore walls of COFs. Additionally, the single-ion sites of this lithium carboxylate salt can also facilitate ion conduction and enhance the lithium-ion transference number. Therefore, we report, for the first time, a single-ion COF heterogeneous catalytic ROCOP of cyclic lactones that is also favorable for electrochemical performance. The catalytic mechanism is that the –COOLi group on the COF activates trace amounts of water in the electrolyte, causing the –OH of water molecules to attack the C=O (in CL or TMC) of the cyclic lactone and generate anionic active species for random copolymerization of the cyclic lactones (Fig. 3a) [41].

Firstly, to select the optimal polymerization temperature and reaction time, we referred to the catalytic efficiency of similar catalysts [42] and chose two polymerization temperatures as research objects: 80 °C (Fig. 3b) and 100 °C (Fig. 3c). At a polymerization temperature of 100 °C with a molar ratio of CL to TMC of 4:1, it can be seen that after 24 h, the conversion of CL and TMC reached 52.8% and 68.3%, respectively, and after 48 h, the conversion both reached over 99.0%. Therefore, in the subsequent in situ polymerization, a polymerization time of 48 h and a reaction temperature of 100 °C were selected as the reaction conditions. To verify whether it is the –COOLi group on the COF or the LiTFSI precursor in the electrolyte that catalyzes, we conducted polymerization experiments without TpPa-COOLi. The ^1^H NMR results of the original solution showed that only LiTFSI cannot catalyze the polymerization of CL and TMC (Fig. 3d). The reason is that although the substantial quantity of Li^+^ dissociated from LiTFSI can be regarded as Lewis acids, potentially facilitating the activation of the more readily polymerizable TMC to initiate ROCOP, the characteristic absorption peaks of carbonyl groups within the range of 1650 to 1800 cm^−1^ in the FTIR spectra of the precursor solution comprising 30 wt% LiTFSI in 4CL-TMC monomers precursor, with an equivalent lithium salt content in the electrolyte, carbonyls were observed to be only coordinated with Li⁺ in CL, whereas TMC remained uncoordinated (Fig. 3e) [36]. This elucidates the reason why LiTFSI is unable to initiate the ROCOP of CL and TMC. For CL, the prerequisites for ring-opening polymerization are more stringent, necessitating the presence of stronger Lewis or protonic acids. Consequently, LiTFSI is ineffective in catalyzing the ROCOP of these two monomers.

**Fig. 4:**
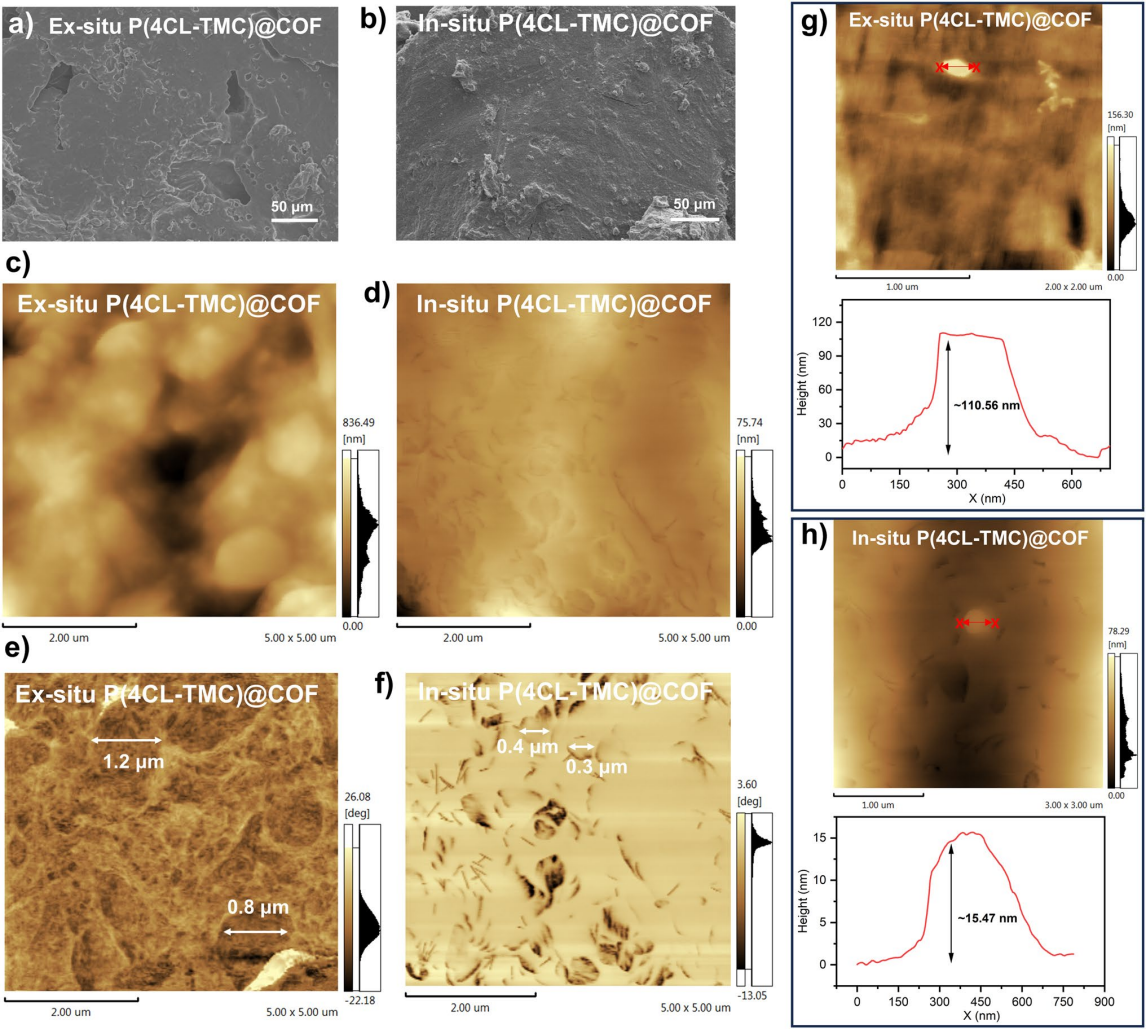
SEM images of surface morphologies of **a** ex situ **b** and in situ P(4CL-TMC)@COF films. AFM bitmap diagram of **c** ex situ and **d** in situ P(4CL-TMC)@COF films. AFM phase diagram of **e** ex situ and **f** in situ P(4CL-TMC)@COF films. AFM image with roughness of **g** ex situ and **h** in situ P(4CL-TMC)@COF films

We employed a synergistic approach utilizing SEM and atomic force microscopy (AFM) to scrutinize the surface morphologies of both ex situ P(4CL-TMC)@COF and in situ P(4CL-TMC)@COF films, along with assessing the dispersion of COF within the polymeric matrix. SEM images revealed the presence of numerous pores and defects on the surface of the polymer film fabricated via ex situ methods attributed to solvent evaporation (Fig. 4a). These imperfections, corresponding to the varying depths in the AFM bitmap (Fig. 4c), could introduce vacancies and elevate interface impedance upon electrode interface interaction, thereby impeding ion transport. Conversely, the in situ synthesized polymer film exhibited a smooth surface with minimal defects (Fig. 4b, d). The AFM phase diagram further elucidated that COF (represented by dark areas) in the ex situ P(4CL-TMC)@COF displayed the serious aggregations (Fig. 4e), whereas in the in situ P(4CL-TMC)@COF, COF was uniformly dispersed, exhibiting thin-layer characteristics (Fig. 4f). In addition, we measured the particle size of the dark regions in the phase diagram, which correspond to the COF particles. The results indicated that in ex situ P(4CL-TMC)@COF, the COF particles tend to aggregate, forming large clusters with a particle size of approximately 1.2 µm. In contrast, in the in situ P(4CL-TMC)@COF, the COF particles are significantly reduction, resulting in a reduced particle size of around 0.4 µm. This observation highlights the distinct structural characteristics of COF particles in the two different synthesis approaches, suggesting that the in situ polymerization process effectively mitigates COF aggregation and promotes a more uniform dispersion of COF particles. Based on these observations, we hypothesize that confinement polymerization within COF pores is facilitated by partial exfoliation of the COF layer, thereby maximizing polymer-COF contact area and ensuring homogeneous COF dispersion.

To validate the hypothesis, we conducted an analysis of the thickness of COFs dispersed within polymers by using AFM. The results showed that the original COF thickness, before blending, was approximately 142.13 nm (Fig. S10). When utilizing ex situ composite methods, the thickness remained relatively unchanged at around 110.56 nm (Fig. 4g). This observation aligns with the aggregation of COFs noted in the phase diagram, suggesting that COFs and polymers primarily form superficial interactions in ex situ P(4CL-TMC)@COF group, which are not conducive to effective COF dispersion. In contrast, within the in situ P(4CL-TMC)@COF group, partial exfoliation of the COF layer did occur (the thickness of the COF monolayer calculated to be 0.17 nm based on the Bragg equation), and the stripped COF thickness measured at 15.47 nm (Fig. 4h). This indicates that the confined polymerization of the monomers within COF pores, facilitated by partial exfoliation, did indeed enhance the contact area between the polymer and COF, thereby improving the dispersion of COF. Following the polymerization within the pores, COF-polymer junctions were formed without destroying the long-range ordered structure of the COF, which is more conducive to the rapid Li^+^ transport along the COF pores wall.

To investigate the impact of the blending method of polymer and COF on Li^+^ transport efficiency, we characterized the physical properties of two distinct blending processes of the two COF-polymer composite materials (LiTFSI was excluded to isolate the influence of lithium salt on the polymer). First, a thermogravimetric analyzer (TGA) was employed to assess the thermal stability, with the curves presented in Fig. S11. The results indicated that the thermal decomposition temperature (*T*_d5_) of P(4CL-TMC), in situ P(4CL-TMC)@COF, and ex situ P(4CL-TMC)@COF was 295, 280, and 234 °C, respectively. Notably, in situ blending facilitates the uniform dispersion of COF in the polymer, reducing phase separation phenomena [43, 44]. Enhanced interfacial interactions can boost the blending materials' overall thermal stability [45, 46]. This observation is consistent with different scanning calorimetry (DSC) results (Fig. S12), which show that the glass transition temperature (*T*_g_) of P(4CL-TMC) is only − 54.5 °C In comparison, both in situ blending (*T*_g_ = − 63.4 °C) and ex situ blending (*T*_g_ = − 56.7 °C) effectively lower the *T*_g_ of the polymer and enhance the flexibility of the polymer segments [45]. This indicates that the in situ composite process is more beneficial for lithium-ion conduction. To further understand the reasons for the difference in glass transition temperature between the in situ P(4CL-TMC)@COF and ex situ P(4CL-TMC)@COF, considering that the copolymer of CL and TMC is also a crystalline polymer, X-ray diffraction (XRD) was used to characterize the crystallization strength of these polymers (Fig. S13). A strong PCL crystallization diffraction peak in the range of 2θ = 17° ~ 25° was observed [36], and it was found that the intensity of the crystallization diffraction peak in the in situ P(4CL-TMC)@COF group was the weakest, indicating that the in situ composite process can more effectively reduce the crystallinity of the polymer. The increased proportion of amorphous regions enhances the mobility of the polymer segments, which is confirmed by DSC results, ultimately improving lithium-ion conduction.

The porosity of the TpPa-COOLi sample was confirmed via nitrogen (N_2_) adsorption/desorption isotherms, yielding a Brunauer–Emmett–Teller (BET) surface area of 75.96 m^2^ g^−1^ at 77 K (Fig. S14). The low surface area of the carboxylic acid COF is attributed to its poor crystallinity. The pore size distribution exhibits characteristics of micropores, with cavity diameters of 1.37, 1.41, and 1.70 nm. The high porosity and uniform pore size distribution of TpPa-COOLi facilitate the migration of lithium ions and the penetration of polymers. Concurrently, a marked decline in the BET-specific surface area was observed for both in situ P(4CL-TMC)@COF and ex situ P(4CL-TMC)@COF groups, suggesting a substantial confinement of polymer segments within the nanopores of COF particles. This notable reduction in both the specific surface area and pore volume underscores the confinement effect, which is poised to expedite Li^+^ dissociation and promote rapid ion transport, thereby further enhancing ion conduction both within and beyond the pore confines.

**Fig. 5:**
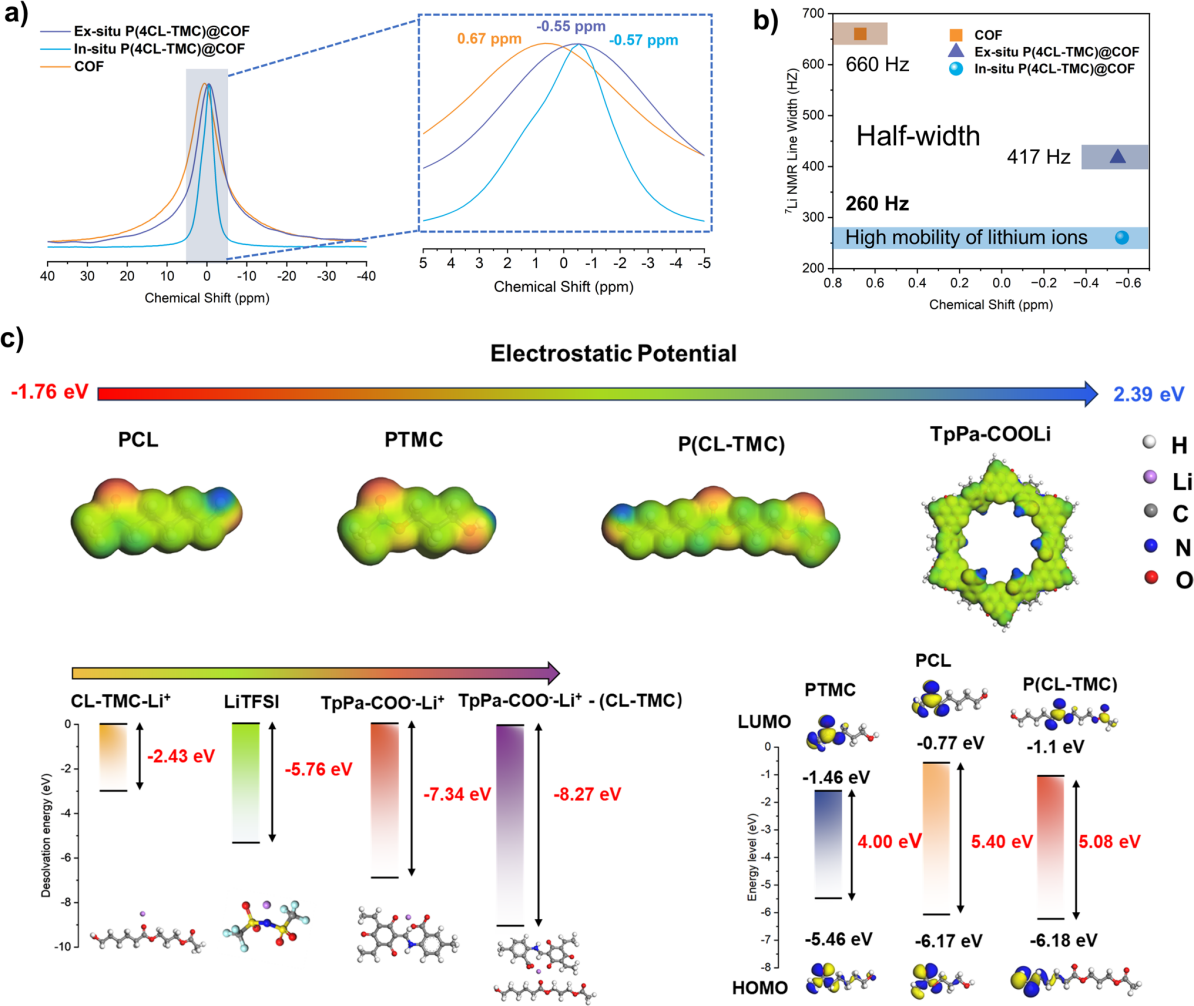
**a** ^7^Li solid-state magic-angle-spinning (MAS) NMR spectra comparison of TpPa-COOLi, in situ P(4CL-TMC)@COF, and ex situ P(4CL-TMC)@COF. **b** The plot of chemical shift versus ^7^Li signal peak half-width curves of TpPa-COOLi, in situ P(4CL-TMC)@COF, and ex situ P(4CL-TMC)@COF. **c** DFT calculations of electrostatic potentials, lithium desolvation energies of COF-polymer composite and energy level of PTMC, PCL, P(CL-TMC)

Furthermore, we aim to delve into the potential interaction between the polymer within the blend material and the lithiated COF, exploring whether in situ blend materials exhibit enhanced Li^+^ transport efficiency, as suggested by the previously analyzed physical properties of the polymer. For this, ^7^Li solid-state MAS NMR spectroscopy was employed to analyze the chemical environment surrounding Li^+^ in all samples (Fig. 5a). As evident from the spectra, the chemical shift of Li^+^ in pure TpPa-COOLi, without any polymer blending post-lithiation (depicted as the yellow COF group), resides at approximately 0.67 ppm. However, in both in situ P(4CL-TMC)@COF and ex situ P(4CL-TMC)@COF, the Li^+^ chemical shift shifts and reaches − 0.55 and − 0.57 ppm, respectively. This downward shift can be attributed to an increase in the electron cloud density around Li^+^ in the –COOLi functional group on COF, stemming from the coordination between C=O groups and Li^+^ within the polymer matrix. This finding indicates that Li^+^ within COF engage in ion conduction through interactions with the contacting polymer and extensive coordination with C = O groups present in the polymer [29]. Notably, regardless of whether the blending is in situ or ex situ, the chemical environment of Li^+^ remains consistent. Subsequently, we quantified the half-width of the Li^+^ signal peak in the ^7^Li solid-state MAS NMR spectra, as this metric reflects the strength of Li–Li dipole interactions and, indirectly, the mobility of Li^+^ within the lattice [47]. A plot of chemical shift versus ^7^Li signal peak half-width was constructed (Fig. 5b). The calculations reveal that the half-width for TpPa-COOLi is 660 Hz, while it decreases to 417 Hz for ex situ P(4CL-TMC)@COF and a minimum of 260 Hz for in situ P(4CL-TMC)@COF. The broader half-width in pure COF suggests hindered Li^+^ mobility, as transport primarily occurs along COF pores, encountering energy barriers at grain boundaries. The incorporation of the polymer facilitates Li^+^ transport across these boundaries, narrowing the half-width.

^7^Li solid-state MAS NMR spectra analysis and computational investigations were conducted to elucidate the ion transport mechanism within COF-polymer composite electrolytes. Density functional theory (DFT) calculations of electrostatic potentials and lithium desolvation energies revealed that oxygen atoms in the polymer matrix exhibit strong electronegativity, facilitating enhanced coordination with Li⁺ ions from COFs. This coordination induces Li^+^ electron cloud densification, consistent with the observed downfield shift in Li NMR spectra following polymer blending. Notably, the desolvation energy for Li⁺-polymer interactions (− 2.43 eV) was significantly lower than that for –COOLi groups in pristine COFs (− 7.34 eV), indicating preferential Li⁺ coordination with polymer C=O groups. This energetically favorable interaction promotes Li⁺ release from COF carboxylate substituents. In situ formed polymer and COF junctions demonstrated substantially narrowed Li⁺ NMR half-peak widths, attributable to increased interfacial contact between COFs and polymer chains that facilitates more efficient Li⁺ liberation. The strongly electrophilic –COOLi substituents preferentially coordinate with TFSI⁻ anions and polymer carbonyl oxygen atoms, effectively restricting anion mobility while enhancing Li⁺ transference numbers through TFSI⁻ immobilization.

**Fig. 6:**
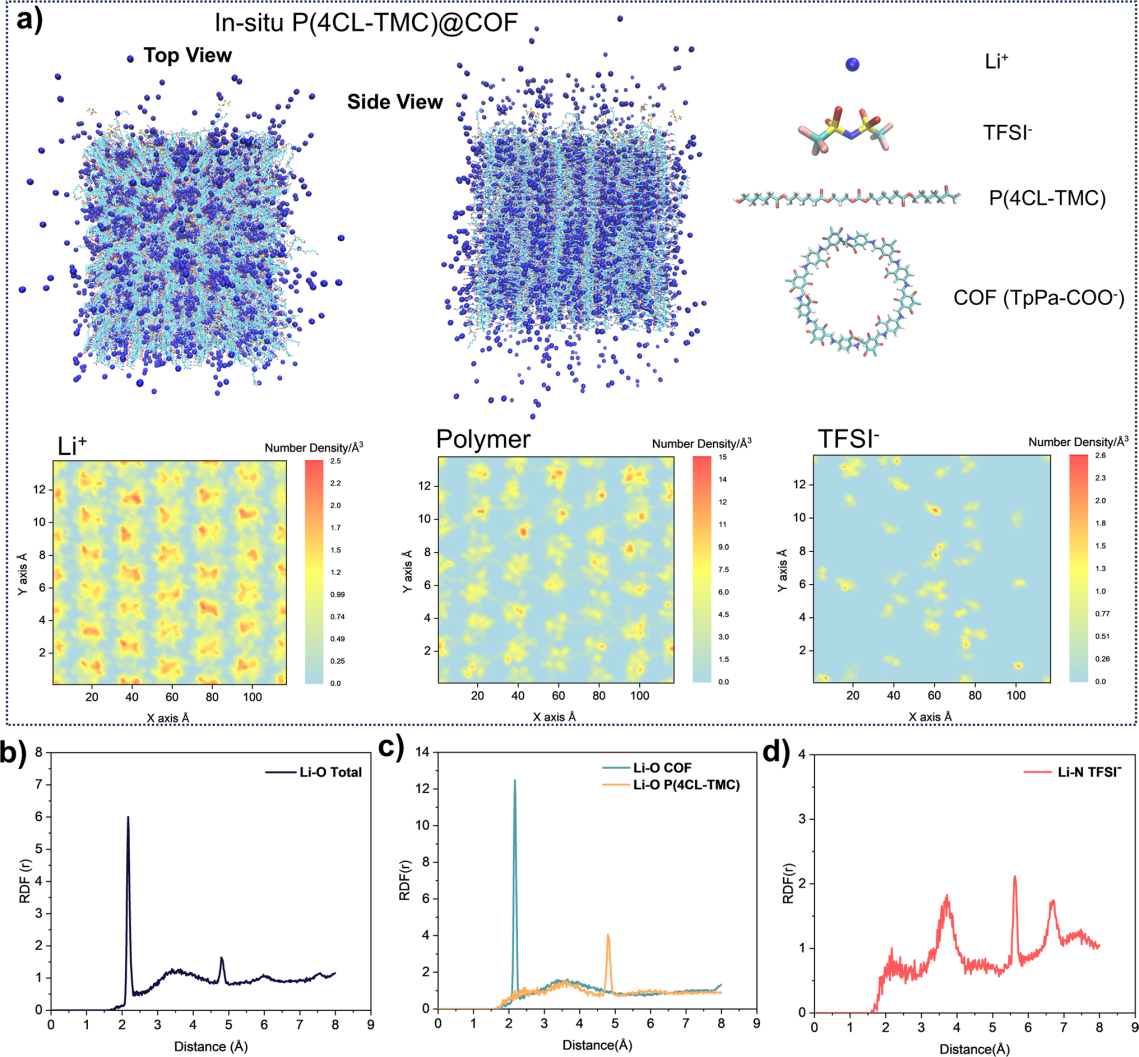
**a** Molecular dynamics simulations for in situ P(4CL-TMC)@COF. Radial distribution function in **b** Li–O (Total COF and Polymer), **c** Li–O (–COOLi in COF) and Li–O (C=O–Li in polymer), and **d** Li–N (Li–N in LiTFSI)

Molecular dynamics simulations (Fig. 6a) revealed distinctive Li–O coordination features in radial distribution functions (RDFs, Fig. 6b–d): two sharp peaks at 0.22 nm (Li⁺-carboxylate coordination) and 0.48 nm (Li⁺-polymer carbonyl coordination), with an intervening broad peak suggesting cooperative ion transport mechanisms. This broad peak corresponds to the desolvation energy of Li^+^ in the TpPa-COO^−^-Li^+^-(CL-TMC) structure as calculated by DFT. After polymer in situ polymerization in COF, more stable and higher desolvation energy (− 8.27 eV) sites are formed compared to TpPa-COOLi alone (− 7.34 eV). This means that lithium ions on TpPa-COOLi can interact well with the polymer, enabling lithium-ion conduction via the carbonyl groups on the polymer backbone. Thus, the COF-polymer composite material has more ion- conduction sites. Two-dimensional number density mapping of the COF-polymer junction architecture showed significant Li⁺ and polymer accumulation within COF nanochannels (high-density red regions in figures), with local concentrations substantially exceeding those of TFSI⁻ anions. This spatial confinement creates efficient Li⁺ conduction pathways along the COF-polymer interface, consistent with RDF analyses. The remarkably low specific surface area of the composite material confirms successful polymer infiltration into COF channels, forming well-defined junction structures.

Comparing in situ and ex situ blending approaches, the in situ composite process results in a more uniform dispersion of COF within the polymer, enlarging the interface area between them and enhancing interfacial interactions. Leveraging COF's vast specific surface area, monomers are sequestered and polymerized within its nanoscale pores, fostering the formation of an in situ polymer-COF junction, as opposed to mere surface interactions in ex situ blends [34]. Through in situ polymerization within the COFs channels, partially exfoliates the COFs and forms more junctions between COFs and the polymer. This not only reduces the large-scale aggregation of COF particles but also mitigates concentration polarization and non-uniform electric field distribution caused by particle agglomeration. As a result, it enables more uniform lithium deposition and fast Li^+^ transportation. Confined polymerization enables Li^+^ within COF pores to coordinate efficiently with C=O groups in the polymer, accelerating Li^+^ transport within the composite by providing dual-pathway transmission and assisting the Li^+^ transport on the COF crystal plane. This aligns with prior analysis highlighting that improving the flexibility of in situ blended polymer-COF segments enhances Li^+^ transport. In conclusion, we contend that in situ blending of COF and polymer for Li^+^ transport surpasses many previous ex situ blending works in terms of efficiency. Consequently, in subsequent electrochemical performance discussion, we adopted in situ composite technology to assess the impact of COF's long-range ordering and the presence of -COOLi substituents on electrochemical performance.

### Electrochemical Performances of Polymer Electrolytes

It is widely acknowledged that the incorporation of COF as a filler can significantly enhance the electrochemical properties of polymers when blended with them [48, 49]. Although the plasticization of polymers by COF and the unique Lewis acid–base or ionic interactions with lithium salts or polymers often serve as auxiliary benefits, there has been limited exploration into how the inherent structural features of COF directly influence the overall electrochemical performance of the composite material. In this work, we have designed and prepared an anionic COF, TpPa-COOLi, which boasts a crystalline structure with long-range order and distinctive –COOLi substituents. To unravel which structural element of TpPa-COOLi contributes to its enhancing effect on electrochemical performance, three distinct COF materials: crystalline TpPa-COOLi, amorphous NCTpPa-COOLi, and TpPa blended with lithium benzoate but devoid of –COOLi substituents in its structural units were also synthesized (Fig. S15). Employing an identical freeze-drying approach with expanded pore cellulose loading, these COFs with a polymer P(4CL-TMC) were in situ blended, named as polymer@TpPa-COOLi, polymer@NCTpPa-COOLi and polymer@TpPa, respectively.

**Fig. 7:**
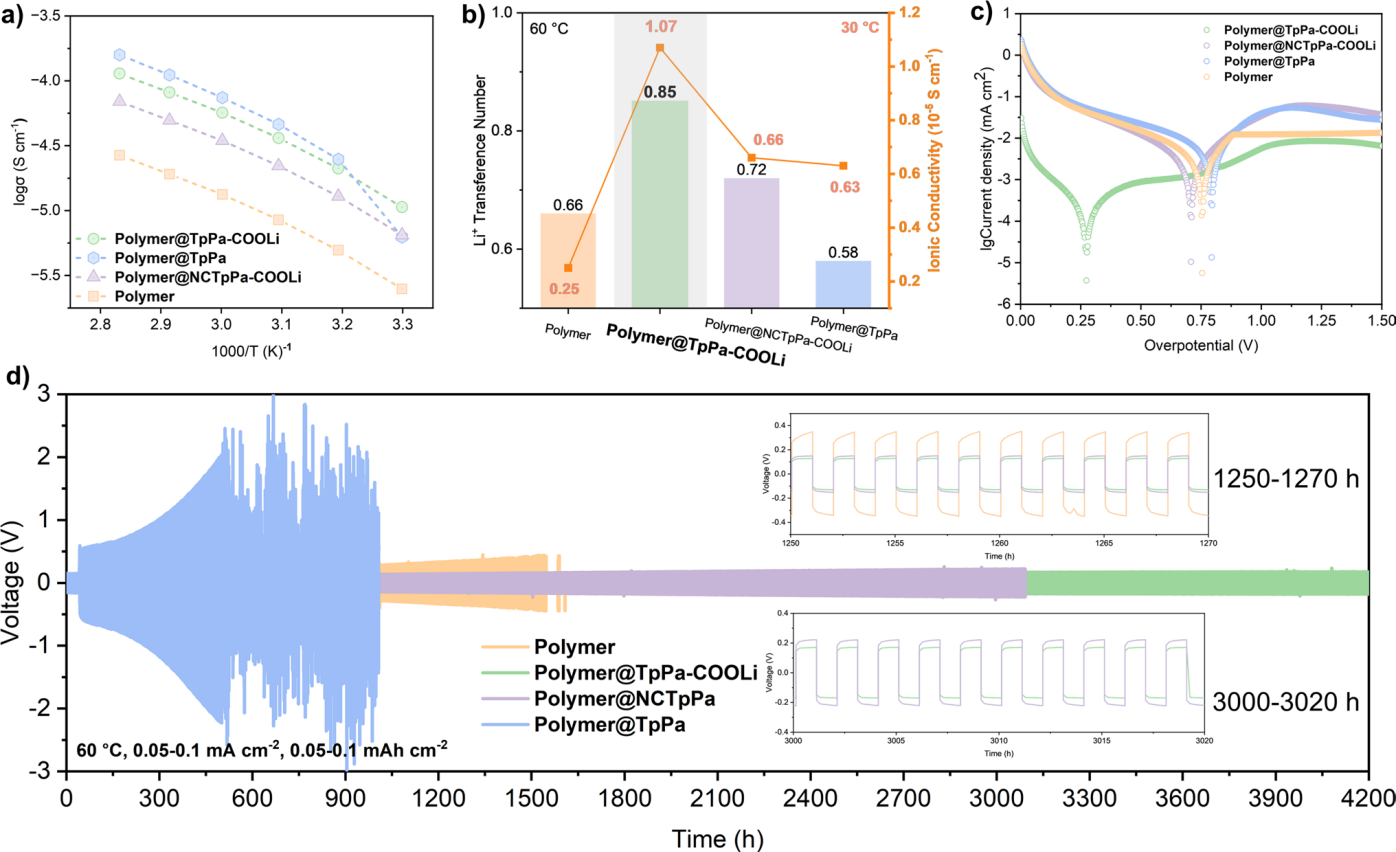
**a** Temperature dependence of ionic conductivity for polymer, polymer@TpPa-COOLi, polymer@NCTpPa-COOLi, and polymer@TpPa. **b** Lithium-ion transference number bar chart for polymer, polymer@TpPa-COOLi, polymer@NCTpPa-COOLi, and polymer@TpPa. **c** Tafel curves of polymer, polymer@TpPa-COOLi, polymer@NCTpPa-COOLi, and polymer@TpPa. **d** Voltage profiles of Li/Li symmetric cell of polymer, polymer@TpPa-COOLi, polymer@NCTpPa-COOLi, and polymer@TpPa with the current density of 0.1 mA cm^−2^ at 60 °C

The experiments on the temperature dependence of ionic conductivity revealed that the pure polymer group P(4CL-TMC) exhibited a relatively low ionic conductivity of 2.5 × 10^−6^ S cm^−1^ at room temperature (Fig. 7a). This is attributed to the weak lithium-ion complexation effect and lithium salt dissociation capability of pure polyester-based polymers, coupled with the fact that PCL is a crystalline polymer where lithium ions can only migrate within the amorphous regions. Upon in situ blending with COF, the ionic conductivity of all groups was significantly enhanced, primarily due to the reduction in PCL crystallinity. Furthermore, the interaction between the C=O groups in the polymer and the –COOLi bound Li⁺ on COF facilitated lithium salt dissociation, thereby increasing the overall carrier concentration. For the blends containing lithium benzoate (polymer@TpPa and the disordered polymer@NCTpPa-COOLi), the room temperature conductivity rose to 6.3 × 10^−6^ and 6.6 × 10^−6^ S cm^−1^, respectively. Notably, the crystalline, long-range ordered TpPa-COOLi exhibited a superior room temperature conductivity of 1.1 × 10⁻^5^ S cm^−1^, attributed not only to the increased Li⁺ carrier concentration but also to the rapid ion conduction facilitated by the porous COF structure [32]. As temperature increases, the mobility of benzoate ions in the dissociated large anion moiety of the blend is enhanced, contributing to the anion conductivity of the material. Consequently, polymer@TpPa displayed the highest conductivity of 7.90 × 10⁻^5^ S cm^−1^ across all groups at 60 °C. However, at this temperature, polymer@TpPa-COOLi and polymer@NCTpPa-COOLi achieved conductivities of 5.62 × 10⁻^5^ and 3.32 × 10⁻^5^ S cm^−1^, respectively, confirming that the long-range ordered porous structure of COF is pivotal in accelerating ion conduction. Despite its superior conductivity, the interfacial stability of benzoate ions as a non-electrolyte component in polymer@TpPa at 60 °C was found to be poor, leading to high interfacial impedance and low critical current density in its lithium symmetric battery (Figs. S16 and S17).

The lithium-ion transference number (*t*_Li_^+^) is paramount in determining lithium deposition performance. A low *t*_Li_^+^ can trigger anion depletion in proximity to the lithium electrode, giving rise to a substantial electric field that fosters lithium dendrite growth [8]. To avert anion depletion, SPEs with superior ionic conductivity and lithium-ion mobility are highly desirable. For the pure polymer P(4CL-TMC), the feeble coordination between ester groups and lithium ions facilitates lithium-ion transport, yielding a high *t*_Li_^+^ of 0.66 (Fig. 7b). Upon incorporating COFs into the polymer matrix, the *t*_Li_^+^ undergoes a further boost. Notably, the crystalline polymer@TpPa-COOLi (*t*_Li_^+^ = 0.85) displays a higher *t*_Li_^+^ than the amorphous polymer@NCTpPa-COOLi by 0.13 (Figs. S18-S25). This disparity stems from the unique in situ polymerization growth approach within the long-range ordered pores of the crystalline variant, which restricts TFSI^−^ anion transport within the pores. A comparative analysis between polymer and polymer@NCTpPa-COOLi underscores that the fixed –COO^−^ groups also contribute positively to the *t*_Li_^+^ value. Conversely, the polymer@TpPa hybrid, which incorporates lithium benzoate and TpPa, exhibits the lowest *t*_Li_^+^ of 0.58. This is attributed to the substantial involvement of dissociated benzoate ions in ion migration. Based on a comprehensive assessment of ionic conductivity and lithium-ion transference numbers (Fig. 7b), the solid polymer electrolyte featuring long-range ordered crystalline pore COF emerges as the most advantageous in terms of lithium-ion transport efficiency.

The implementation of SPEs featuring a wide electrochemical stability window (ESW) serves as a cornerstone for ensuring stable battery cycling performance [50, 51]. In this investigation, the oxidation decomposition potential of the composite polymer electrolyte was rigorously examined employing linear sweep voltammetry (LSV). The results demonstrate the remarkable ESWs exhibited by the prepared SPEs, as illustrated in Fig. S26. Notably, the polymers based on PCL and PTMC matrices inherently possess elevated electrochemical stability, contributing to the exceptional performance of the four electrolyte systems which exhibit high oxidation decomposition potentials of 5.06, 5.26, 5.25, and 5.18 V, respectively (Fig. S27). These findings underscore the substantial potential of SPEs to accommodate high-voltage cathodes.

### Electrochemical Performances of Lithium Batteries

The Tafel plot shown in Fig. 7c illustrates that polymer@TpPa-COOLi exhibits the highest exchange current density within the group, specifically 3.11 × 10^–4^ mA cm^−2^. In contrast, NCpolymer@TpPa-COOLi displays an exchange current density of 4.55 × 10^–3^ mA cm^−2^, which is 6.84 times greater (Fig. S28), thereby demonstrating that the ion transport performance following partial exfoliation surpasses that of the amorphous COF. Concurrently, among these four groups, polymer@TpPa-COOLi possesses the lowest polarization overpotential of 0.27 V, indicative of minimal polarization within the electrolyte. To further scrutinize the current-handling capability of these electrolytes, lithium symmetric cells were fabricated and subjected to lithium deposition/stripping at varying current densities. As depicted in Figs. S16 and S17, polymer@TpPa-COOLi and polymer@NCTpPa-COOLi stand out due to their exceptional conductivity and high lithium-ion transference number, enabling a critical current density of 0.50 mA cm⁻^2^ markedly surpassing the 0.35 mA cm⁻^2^ achieved by the reference polymer group. While the polymer@TpPa group boasts the highest conductivity among the samples, the incorporation of non-electrolyte lithium benzoate component has a detrimental effect on interfacial compatibility, ultimately leading to a reduced critical current density 0.15 mA cm⁻^2^ and an elevated overpotential. This observation underscores the intricate interplay between electrolyte composition and interfacial properties in determining overall battery performance.

The cycling performances of four sets of polymer-based lithium symmetric batteries are depicted in Fig. 7d. Notably, the over potential of all four assembled Li//Li batteries exhibits a gradual increase over time. Leveraging the exceptional interface compatibility afforded by in situ polymerization technology, the polymer group notably sustains a prolonged cycle duration of 1500 h at a current density of 0.1 mA cm⁻^2^. However, a significant short circuit ensues due to the excessive proliferation of lithium dendrites puncturing the separators. The polymer@TpPa group initially displays a continuous escalation in over potential, suggesting that the persistent side reactions between lithium benzoate and lithium metal at the electrolyte interface contribute to an increase in interfacial impedance. In contrast, polymer@TpPa-COOLi, at a current density of 0.1 mA cm⁻^2^, exhibits an impressive cycling time of 4200 h and maintains stable cycling for over 800 h even at 0.2 mA cm⁻^2^ (Figs. S29–S31). This enhanced performance stems from the more stable interface contact within the system, coupled with high conductivity and *t*_Li_^+^ values. Polymer@NCTpPa-COOLi, when compared to the polymer@TpPa-COOLi, underscores the significant role of long-range ordered pores in COF, which markedly enhance the transport efficiency of Li^+^ in the polymer.

**Fig. 8:**
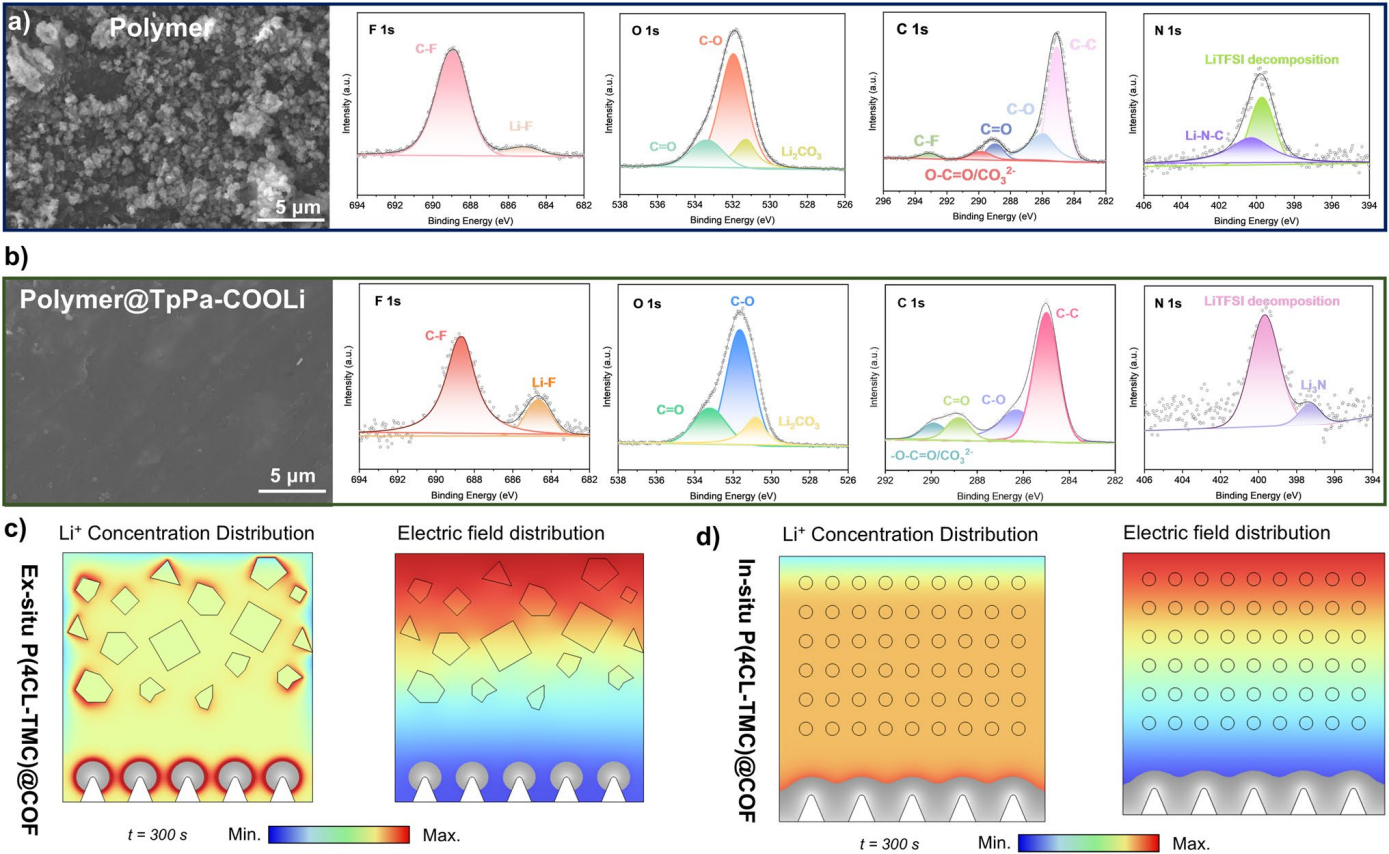
**a** SEM image and XPS spectra of F 1*s*, C 1*s*, O 1*s*, and N 1*s* of Li surface after cycling 1500 h from Li/Polymer/Li. **b** SEM image and XPS spectra of F 1*s*, C 1*s*, O 1*s*, and N 1*s* of Li surface after cycling 4200 h from Li/Polymer@TpPa-COOLi/Li. Finite element analysis to simulate the lithium-ion concentration, electric field distribution, and lithium dendrite growth of **c** ex situ P(4CL-TMC)@COF and **d** in situ P(4CL-TMC)@COF

Further examination of Li was conducted using SEM on the anode surface topography of Li after cycling for 4200 h in Li/Polymer@TpPa-COOLi/Li and after cycling for 1500 h in Li/Polymer/Li. In the anode surface of the polymer group exhibited uneven protrusions, minute cracks, and pronounced dendritic growth (Fig. 8a). Conversely, polymer@TpPa-COOLi group, the lithium surface displayed a smooth and flat morphology without evident dendritic growth (Fig. 8b). These results indicate that when utilizing materials with enhanced ion transport efficiency, such as polymer@TpPa-COOLi in the electrolyte, the polymer component within the lithium-ion solvated sheath is reduced, and its binding capacity to lithium ions is weaker. This is attributed to the synergistic transport of lithium ions facilitated by both COF and the polymer.

XPS elemental analysis was conducted on the surface topography of the lithium anode after cycling for 4200 h in Li/Polymer@TpPa-COOLi/Li and after cycling for 1500 h in Li/Polymer/Li configurations. According to the F 1*s* spectrum, it can be deduced that at 684.6 eV, polymer@TpPa-COOLi exhibited more characteristic peaks of LiF, whereas only weak LiF signals were detected in the polymer group. This suggests that in polymer@TpPa-COOLi, the electrolyte contains a higher concentration of TFSI^−^ in the primary solvation structure surrounding rapidly transported Li^+^, leading to the decomposition and formation of additional inorganic SEI components. In contrast, solvation in the polymer group primarily relies on the coordination of the polymer with lithium ions, with concurrent decomposition of the polymer and TFSI^−^ anions resulting in feebler organic C-F SEI components at 688.7 eV. This observation aligns with the findings in the N 1*s* spectrum, where at 400.3 eV, the polymer group generated more organic SEI structures composed of Li–N–C components, whereas polymer@TpPa-COOLi produced some Li_3_N at 398.1 eV (Fig. 8b). This indicates that during cycling, polymer@TpPa-COOLi forms an organic–inorganic composite SEI layer, which ensures a more stable electrode–electrolyte interface between the polymer and the anode.

Based on the differences in thickness, morphology, and dispersion of COFs particles in polymer electrolytes prepared via in situ and ex situ blending methods, we employed finite element analysis to simulate the lithium-ion concentration, electric field distribution, and lithium dendrite growth during a certain period of lithium deposition in the electrolyte (Fig. 8c, d). The results indicate that in the in situ P(4CL-TMC)@COF electrolyte, COFs particles achieve uniform dispersion due to the formation of junctions with the polymer. This uniformity prevents lithium ions from aggregating on a particular surface, leading to a homogeneous lithium-ion concentration field and minimal concentration polarization (Fig. 8d). Consequently, lithium dendrites grow more uniformly. In contrast, in the ex situ P(4CL-TMC)@COF electrolyte, lithium ions tend to accumulate at the interface between the polymer electrolyte and COFs. This accumulation results in significant concentration polarization, causing lithium dendrites to grow irregularly with a propensity for tip growth. A homogeneous ion concentration and electric field distribution effectively suppresses the uncontrolled growth of lithium dendrites while preventing dead Li formation. This mechanism aligns with the experimental observations from prolonged lithium deposition cycling tests in the in situ blended COF-polymer composite system Both the NCTpPa-COOLi@Polymer and TpPa-COOLi@Polymer systems demonstrated exceptional stability, sustaining lithium deposition for over 3000 h without significant short circuit.

**Fig. 9:**
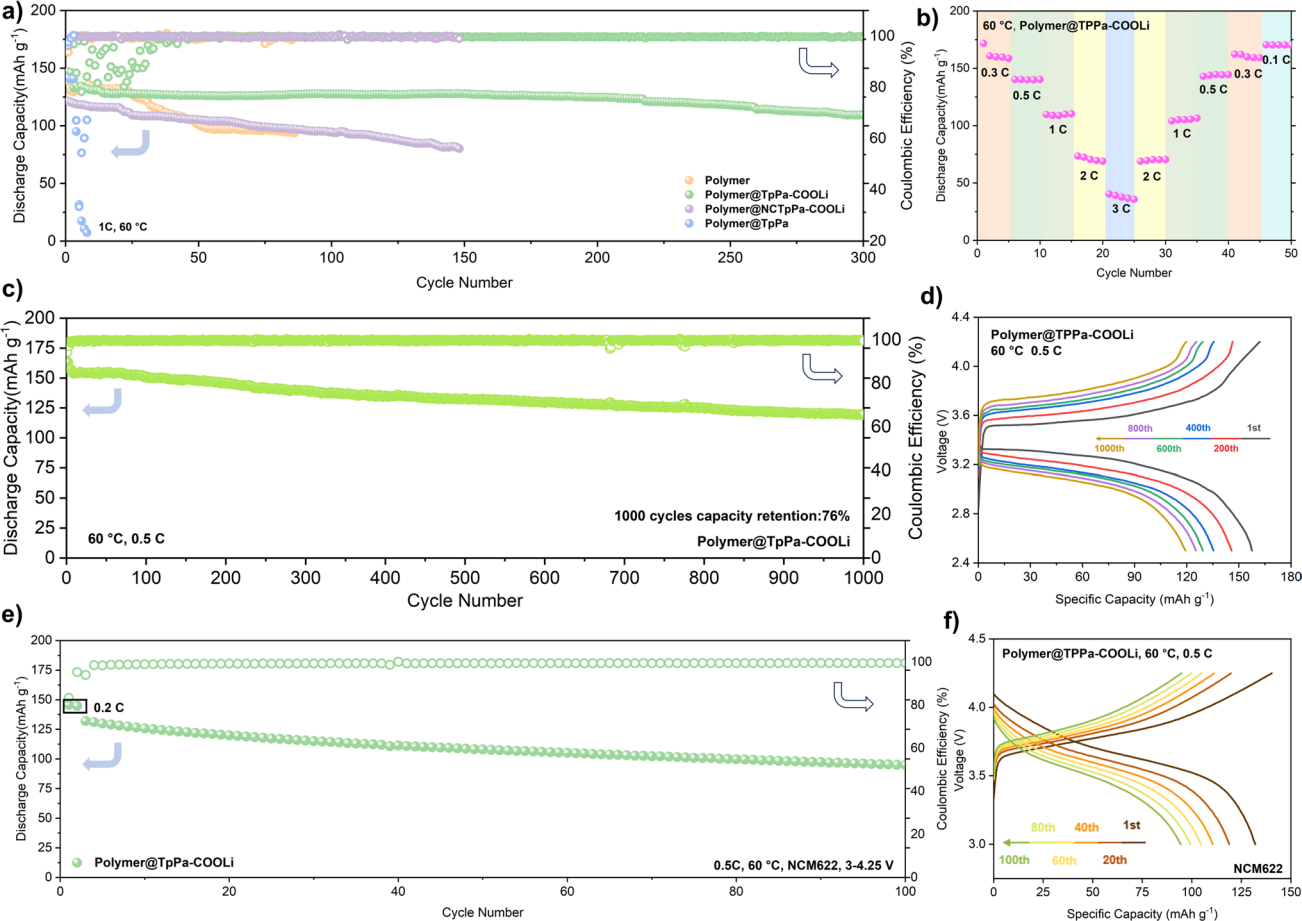
**a** Cycle performance of Li//LFP of polymer, polymer@TpPa-COOLi, polymer@NCTpPa-COOLi, and polymer@TpPa electrolyte at 1C under 60 °C. **b** Specific capacities of polymer@TpPa-COOLi at 0.1C, 0.3C, 0.5C, 1C, 2C, and 3C under 60 °C. **c** Cycle performance and **d** charge–discharge plot of Li/polymer@TpPa-COOLi/LFP at 0.5C under 60 °C. **e** Cycle performance of Li/polymer@TpPa-COOLi/NCM622 at 0.5C under 60 °C. **f** Specific capacities verse voltage curves of Li//NCM622 half-cells of polymer@TpPa-COOLi at 0.5C

An LMB with LiFePO_4_ (LFP) as the cathode was assembled in situ for the purpose of assessing the long-term cycling stability of these in situ composite COF and polymer. Three sets of in situ blended composite electrolytes, comprising polymer@TpPa-COOLi, polymer@NCTpPa-COOLi, and polymer@TpPa, underwent cross flow charging and discharging test conditions at 60 °C and 1C (170 mAh g^−1^) (Figs. 9a, b and S32). The initial discharge specific capacities observed were 132.9, 121.4, and 141.6 mAh g^−1^, respectively, with the polymer group yielding the lowest capacity at 129.4 mAh g^−1^. This disparity is rooted in the previously elucidated benefits of COF pores in facilitating ion transport, thereby enhancing the utilization rates of positive electrode active materials.

Specifically, polymer@TpPa, as indicated by critical current density (CCD) analysis (Fig. S16), exhibited poor stability of lithium benzoate at the Li anode interface, resulting in rapid capacity fade at 1C. After 8th cycles, its capacity dwindled to merely 7.4 mAh g^−1^. In the absence of the long-range ordered structure of COF, polymer@NCTpPa-COOLi electrolytes, which trail only polymer@TpPa-COOLi in terms of their properties, demonstrated stable cycling for 150 cycles at 1C with a capacity retention rate of 66%. Nevertheless, the reliance on polymer for Li⁺ solvation, in the absence of COF pore wall-assisted ion transport, led to an unstable organic SEI interface originating from polymer decomposition. Polymer@TpPa-COOLi, benefiting from the high utilization rate and superior dispersion of COF, exhibited exceptional cycling stability, maintaining a capacity retention rate of 82% after 300 cycles at 1C. Furthermore, it demonstrated a remarkable initial specific capacity of 157.9 mAh g^−1^ at 0.5C, with a capacity retention rate of 76% after 1000 cycles (Fig. 9c, d). This robust performance is attributed to the effective dispersion of COF during in situ blending and the formation of a stable interface with the electrode by using in situ polymerization. Indeed, polymer@TpPa-COOLi electrolyte is well-suited for applications involving high-voltage cathode materials. The electrochemical float test confirmed an oxidation stability of up to 5.0 V for NCM622 cathode material with polymer@TpPa-COOLi (Figs. 9e and S33). The assembled Li/polymer@TpPa-COOLi/NCM622 half battery, cycled at a rate of 0.5C, delivered an initial discharge specific capacity of 132 mAh g^−1^, with a capacity retention rate of 72% after 100 cycles. This underscores the advanced nature and high performance of polymer@TpPa-COOLi in electrochemical applications.

**Fig. 10:**
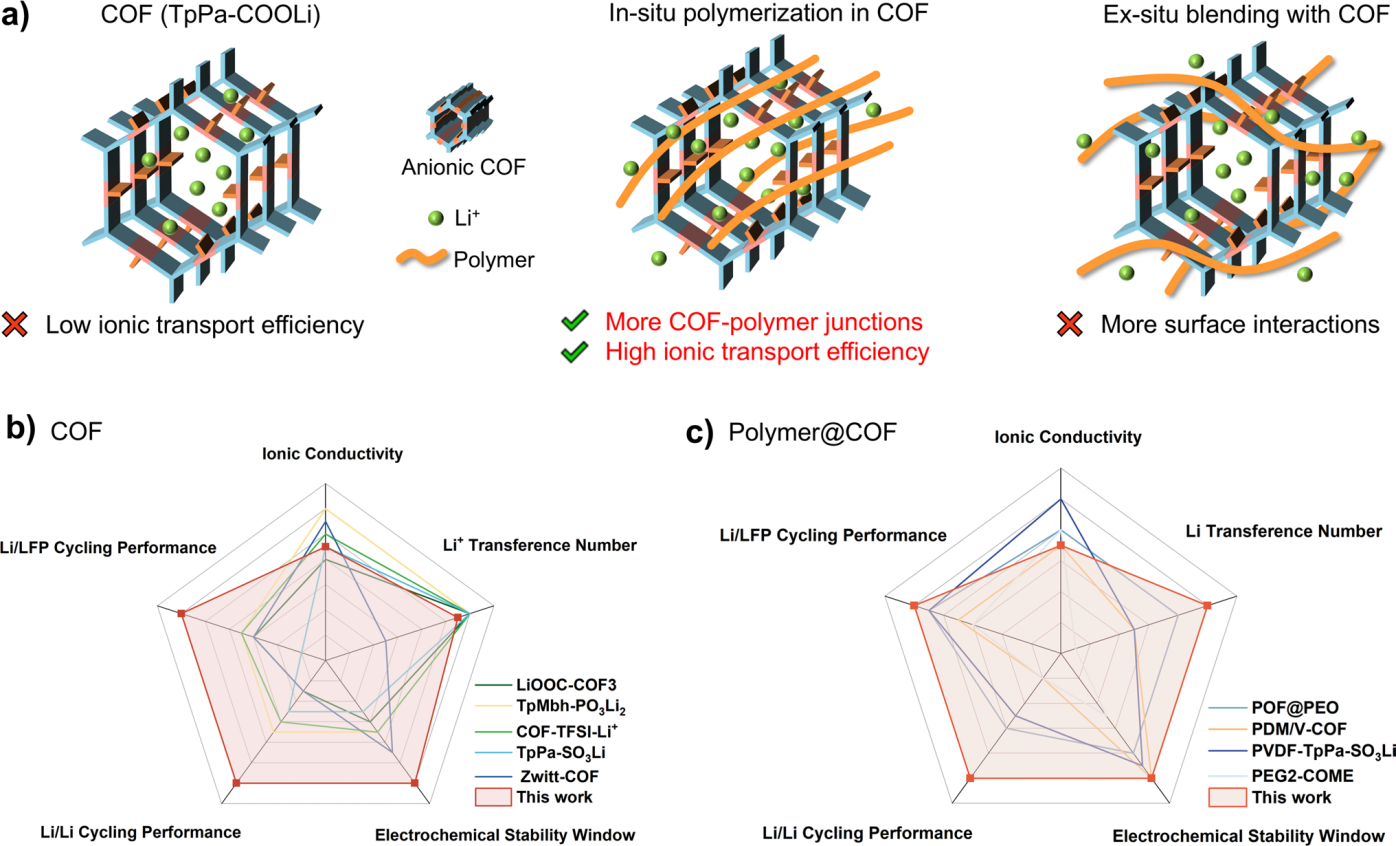
**a** Structure diagrams of COF, in situ formed polymer@COF, and ex situ formed polymer@COF. Radar chart comparing the performance of this work with that of some previously reported works about **b** COF electrolytes and **c** polymer@COF electrolytes

In the majority of researches, researchers concerning pure COF solid electrolytes on devising novel chemical structures aimed at augmenting the dissociation degree of lithium ions within COFs, thereby enhancing the ion transport efficiency (Fig. 10a) [26, 52–55]. Typically, pure COF solid electrolytes pellets are fabricated via hot or cold pressing procedure with or without adhesives, with the objective of mitigating ion transport resistance at COFs grain boundaries through the application of pressure. Nevertheless, assembled batteries still necessitate specialized pressure-maintenance device to guarantee optimal interface contact. Furthermore, COF pellets procured through this ex situ preparation methodology harbor a substantial quantity of voids and defects on the lithium metal surface, leading to a notable interface impedance. Despite potentially extremely rapid ion transport efficiency within the electrolyte, the large interface resistances result in poor performance of assembled Li/LFP half-cells and Li symmetric cells due to this “shortboard effect” upon lithium metal contact [56–59].

Here, our study effectively addresses this “shortboard effect” through in situ polymerization, thereby significantly enhancing the cycling performance of Li/LFP half-cells and Li symmetric cells (Fig. 10b). Undoubtedly, the integration of solid polymers and COF composites represents the optimal approach to ameliorate ion transport impedance at grain boundaries and electrode–electrolyte interface impedance. Yet, traditional ex situ composites have failed to exploit the distinctive large surface area pore structure of COF. Long-chain polymers struggle to penetrate these pores, instead forming mere surface interactions with the polymer, which leads to uneven COF dispersion also the composite electrolyte membrane has many surface defections by using ex situ solvent evaporation. Consequently, the in situ polymerization technique employed in this study effectively facilitates ion conduction at COF grain boundaries and electrolyte–electrode interface. The COF-polymer junctions formed in situ partially exfoliates the COF layers, preserving a degree of long-range order while improving the uniformity of COF dispersion within the polymer. Additionally, it leverages monomers external to the pores to establish a favorable interface for in situ polymerization following wetting of the electrode surface. Owing to the excellent electrode–electrolyte interface contact, this study demonstrates remarkable cycling stability compared to certain ex situ polymer-COF blends (Fig. 10c). Despite these advancements, the current COF preparation process, involving complex multi-step acid–base exchanges, still presents challenges for large-scale production. Our future work will focus on simplifying synthetic methods and addressing cost and feasibility issues to enable broader applications.

## Conclusions

In this work, we propose a single-ion COF catalyst tailored for the ROCOP of lactone monomers. Leveraging the COF's high specific surface area, it efficiently adsorbs the monomer precursor and employs the -COOLi single-ion site to facilitate in situ confinement polymerization within its pore. This in situ confined polymerization technique results in partial exfoliation of the COF and thereby increases the contact interface between the COF and the polymer, significantly enhancing its dispersibility within the polymer matrix. The partially exfoliation retains the ion channels, which accelerating the ion transport efficiency of the polymer. By adopting in situ polymerization technology for polymer electrolytes, we have mitigated the high interfacial impedance of COF-based all-solid-state electrolytes and improved the cyclic stability of their interfaces. The assembled Li symmetric battery demonstrates stable cycling for 4200 h at a current density of 0.1 mA cm⁻^2^. Additionally, the Li//LFP half-cell exhibits an impressive initial specific capacity of 157.9 mAh g^−1^ at a rate of 0.5C, maintaining a capacity retention rate of 76% after 1000 cycles. The in situ confinement polymerization method in COF pores substantially improves pore utilization and mitigates COF aggregation in SPEs, thereby fostering the development of sustainable rechargeable batteries.

## Supplementary Information

Below is the link to the electronic supplementary material.

Supplementary file1 (DOCX 5757 kb)
