# Supplementary material for: In Situ Polymerization in COF Boosts Li-Ion Conduction in Solid Polymer Electrolytes for Li Metal Batteries
Source: Nanomicro Lett. 2025 May 6;17:248. doi: 10.1007/s40820-025-01768-3 (PMC12055722; doi:10.1007/s40820-025-01768-3)
Supplement: Supplementary file 1 — Supplementary file1 (DOCX 5757 kb) [file 40820_2025_1768_MOESM1_ESM.docx]

Supporting Information for

**In-Situ Polymerization in COF Boosts Li-Ion Conduction in Solid Polymer Electrolytes for Li Metal Batteries**

Junchen Meng^1^, Mengjia Yin^1^, Kairui Guo^1^, Xingping Zhou^1^*, and Zhigang Xue^1^*

^1^ Key Laboratory of Material Chemistry for Energy Conversion and Storage, Ministry of Education, Hubei Key Laboratory of Material Chemistry and Service Failure, School of Chemistry and Chemical Engineering, Huazhong University of Science and Technology, Wuhan 430074, P. R. China

*Corresponding authors. E-mail: [zgxue@mail.hust.edu.cn](mailto:zgxue@mail.hust.edu.cn) (Zhigang Xue); [xpzhou@mail.hust.edu.cn](mailto:xpzhou@mail.hust.edu.cn) (Xingping Zhou)

**S1 Experimental Section**

**S1.1 Materials**

2,5-Diaminobenzoic acid (DAA), p-phenylenediamine (Pa), 2,4,6-trihydroxy-benzene-1,3,5-tricarbaldehyde (Tp), ε-caprolactone (CL), trimethylene carbonate (TMC), glacial acetic acid, mesitylene, 1,4-dioxane, lithium iodide, lithium benzoate, THF, DMF, dichloromethane, and acetone were purchased from Innochem used as received without further purification. Lithium bis(trifluoromethanesulfonyl) imide (LiTFSI) and LiOH·H_2_O were purchased from Aladdin used as received without further purification. The cellulose membranes (TF4030, Japanese NKK,$d=30 \mu m$,$\phi=16 mm$) and the batteries (CR-2032 type) were purchased from Saibo electrochemical materials website in Taobao. PVDF, lithium iron phosphate (LiFePO_4_, bulk density of 0.30 g cm^-1^, tap density of 0.8 g cm^-3^, compaction density of 2.0 g cm^-3^, specific surface area of 9.0±3.0 m^2^ g^-1^), Super P, and *N*-methyl-2-pyrrolidone (NMP) were purchased locally.

**S1.2 Preparation of COF**

**S1.2.1 Preparation of TpPa-COOH**

Refer to the synthesis method in the literature [S1]. Pyrex tube was charged with Tp (63.0 mg, 0.3 mmol) and DAA (68.5 mg, 0.45 mmol). Then, a mixed 3.0 mL solution of mesitylene and 1,4-dioxane (1:1 vol) was added into the tube. Subsequently, the tube was sonicated for 10 mins, and CH_3_COOH (0.4 mL, 6.0 M) was added to the mixture. The tube was frozen in a liquid N_2_ bath and sealed under vacuum. After sealing, the tube was placed in an oven at 120 °C for 3 days. After cooling at room temperature, the yielded solid was collected by filtering and washed with DMF, THF and acetone several times. The obtained solid was immersed in anhydrous acetone for 1 day; the solvent was replaced four times per day with fresh solvents. Finally, the active sample was dried at 100 °C for 12 h under vacuum to yield a red powder. All COFs are manually ground using a mortar before use, and then finely ground using a planetary grinder.


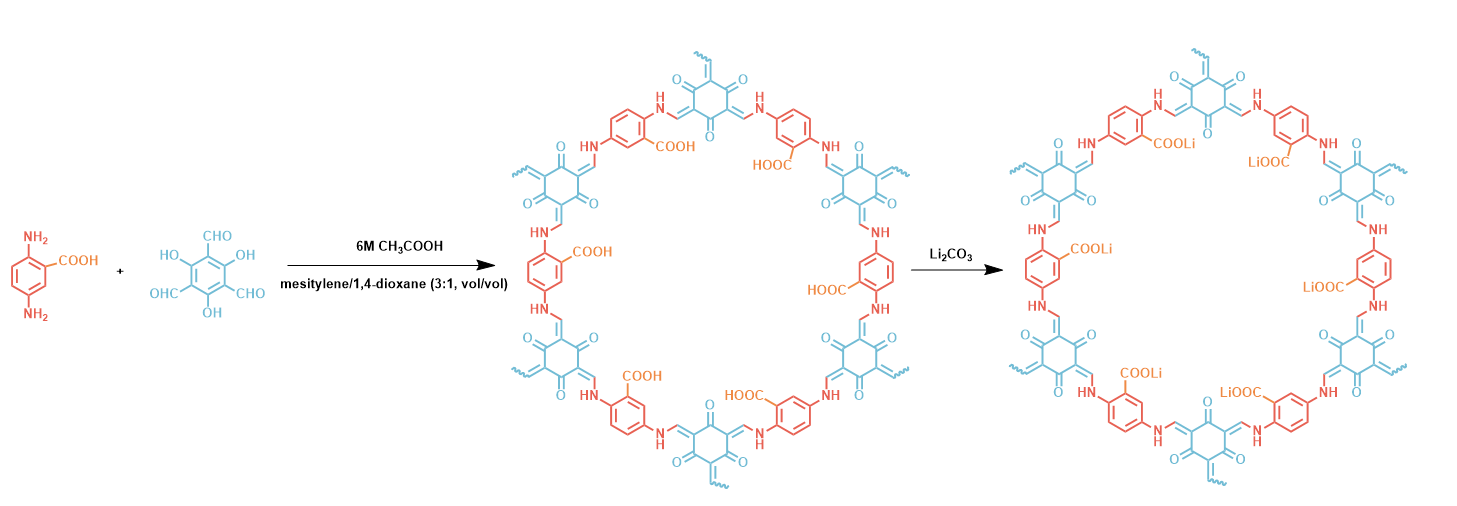


**Fig. S1** Route for the synthesis of TpPa COOLi from DAA and Tp

***S1.2.2 Preparation of TpPa-COOLi***

Refer to the synthesis method in the literature [S1]. In a typical process, the above-synthesized TpPa-COOH (100.0 mg) were suspended in a 20 mL aqueous solution of Li_2_CO_3_ (1 g, 6 M) and stirred at 55 °C 72 h in a round-bottom flask. Subsequently, the resultant solid was collected by filtration and washed with deionized water several times to remove excess Li_2_CO_3_. Finally, the solid was dried at 100 °C for 1 day under vacuum to yield pure TpPa-COOLi. All COFs are manually ground using a mortar before use, and then finely ground using a planetary grinder.

***S1.2.3 Preparation of NCTpPa-COOLi***

Mixing LiOH·H_2_O with DAA in a 1:1 molar ratio in deionized water to obtain lithiated DBA-Li. Then freeze-drying removes water from the solution to obtain DBA- Li. After that Pyrex tube was charged with Tp (63.0 mg, 0.3 mmol) and DAA-Li (72.5 mg, 0.45 mmol). Then, a mixed 3.0 mL solution of mesitylene and 1,4-dioxane (1:1 vol) and 2.5 mL DMF was added into the tube. Subsequently, the tube was sonicated for 10.0 min, and CH_3_COOH (0.4 mL, 6.0 M) was added to the mixture. The tube was frozen in a liquid N_2_ bath and sealed under vacuum. After sealing, the tube was placed in an oven at 120 °C for 3 days. After cooling at room temperature, the yielded solid was collected by filtering and washed with DMF, THF and acetone several times. The obtained solid was immersed in anhydrous acetone for 1 day; the solvent was replaced four times per day with fresh solvents. Finally, the active sample was dried at 100 °C for 12 h under vacuum to yield a red powder. All COFs are manually ground using a mortar before use, and then finely ground using a planetary grinder.

***S1.2.4 Preparation of TpPa***

Refer to the synthesis method [S2] in the literature. After that Pyrex tube was charged with Tp (63 mg, 0.3 mmol) and Pa (49 mg, 0.45 mmol). Then, a mixed 3.0 mL solution of Mesitylene and 1,4-dioxane (1:1 vol) was added into the tube. Subsequently, the tube was sonicated for 10 mins, and CH_3_COOH (0.4 mL, 6.0 M) was added to the mixture. The tube was frozen in a liquid N_2_ bath and sealed under vacuum. After sealing, the tube was placed in an oven at 120 °C for 3 days. After cooling at room temperature, the yielded solid was collected by filtering and washed with DMF, THF and acetone several times. The obtained solid was immersed in anhydrous acetone for 1 day; the solvent was replaced four times per day with fresh solvents. Finally, the active sample was dried at 100 °C for 12 h under vacuum to yield a red powder. Afterwards, the dried powder TpPa and lithium benzoate (57.6 mg, 0.45 mmol) obtained will be dissolved and dispersed in deionized water, and then the water in the solution will be freeze-dried to obtain TpPa powder blended with lithium benzoate. All COFs are manually ground using a mortar before use, and then finely ground using a planetary grinder.

**S1.3 Preparation of P(4CL-TMC)**

CL (4 g, 35 mmol), TMC (0.893 g, 8.75 mmol), and lithium benzoate (56 mg, 0.4375mmol) were ultrasonically dispersed in a 5 mL THF solution in a round bottom flask under argon atmosphere for 10 mins, and then polymerized at 100 ℃ for 48 h. After the reaction is complete, add an additional 5 mL THF to solve the solid, and then precipitate the polymer in dichloromethane, rinse the polymer several times with dichloromethane and deionized water, the white solid was dried at 80 °C for 48 h under vacuum.

**S1.4 Preparation of Polymer@COF**

***S1.4.1 Preparation of In-situ P(4CL-TMC)@COF***

The term COF here refers to TPPa-COOLi. TPPa-COOLi (36 mg, 13 wt% of CL and TMC) were ultrasonically dispersed in CL (0.2 g, 1.748 mmol) and TMC (0.045 g, 0.437 mmol) mixed solutions for 30 mins. After that, stir the solutions vigorously at 100 ℃ for 48 h. The dark red solid was the In-situ P(4CL-TMC)@COF.

***S1.4.2 Preparation of Ex-situ P(4CL-TMC)@COF***

The term COF here refers to TPPa-COOLi. 0.15 g TPPa-COOLi was sonically dispersed in 5 mL THF for 10 min and 1g P (4CL-TMC) was also dissolved in 5 mL THF, respectively. After mixing the two solutions, sonicate for 30 mins. Then pour the mixed solution into the PTFE mold and dry in an 80 ℃ vacuum oven for 48 hs. The dark red solid was the Ex-situ P(4CL-TMC)@COF(13 wt% COF).

**S1.5 Preparation of Polymer@COF electrolyte**

The term COF here refers to TpPa-COOLi, NCTpPa-COOLi, TpPa. The electrolyte preparation and the cell assembly were implemented in an argon gas-filled glove box in which the concentration of O_2_ and H_2_O was below 0.1 ppm. The polymerization precursor comprised was 25 mg LiTFSI and 5mg LiI dissolved in CL (0.2 g, 1.7.5 mmol) and TMC (0.045 g, 0.437 mmol) mixed solutions. The precursor was injected to the correspondingly cellulose separators loaded COF (TpPa-COOLi, NCTpPa-COOLi, TpPa) and assembled with corresponding electrodes in CR-2032 type cells.

**S1.6 Preparation of COF@Cellulose**

In a typical process, disperse 0.1 g TpPa-COOLi in 8 mL deionized water by ultrasound for 30 mins to obtain a stable dispersion. Then add the dispersed droplets onto the surface of the cellulose separators and freeze it in the refrigerator at a temperature of -20 ℃ for 1 h. Place the frozen separators into a freeze dryer with a vacuum degree of 0.1 Pa and a working temperature of -65 ℃. After 8 h of freeze drying, a cellulose membrane loaded on one side was obtained, and then repeat the above operation on the other side to obtain the final cellulose separators loaded COF on both sides.

**S2 Characterization**

The characterization of COF structure was tested by Fourier transform infrared spectroscopy (FT-IR) spectra with ATR mode of the FT interferometer (Equinox 55, Bruker, Germany) over the range of 4000-400 cm^-1^. NMR spectra were measured with deuterium CDCl_3_ as the solvent by a Bruker AV400 NMR machine. Solid-state nuclear magnetic resonance (ssNMR) was performed with a Bruker Avance III 500 MHz wide-bore spectrometer (Germany) with a 194.6 MHz of Larmor frequency for ^7^Li. The ^7^Li spectra with a single-pulse experiment using a π/12 pulse length of 2 μs were referenced to 1 M LiCl (aq) at 0 ppm and recorded using a 4 mm static NMR probe. Reported line widths corresponding to the full width at half-maximum intensity (fwhm) of the spectral peak were measured at room temperature. The crystal structure in COFs was measured by X-ray diffraction (XRD, X'Pert PRO, PANalytical BV, The Netherlands) using Cu Kα radiation (40 mA/40 kV) in the 2θ range of 10-50°. Thermogravimetric analysis (TGA) testing was measured from 30 °C to 800 °C at a heating rate of 10 °C min^-1^ under a nitrogen atmosphere by a 4000 PerkinElmer. Different scanning calorimetry (DSC, Q2000, TA) was measured under N_2_ with a ramp rate of 5 °C min^−1^ from -80 °C to 120 °C. The morphology information of the separators was conducted by scanning electron microscopy (SEM, NovaNanoSEM 450). X-ray photoelectron spectroscopy (AXIS-ULTRA DLD-600W) was carried out to detect the elemental composition of the surface of lithium anode in the cycled symmetric Li//Li cell. The obtained spectra were fitted using Avantage software.

**S3 Electrochemical Measurements**

The electrochemical impedance (EIS) analysis was used to test the ionic conductivities of the in-situ cells assembled with symmetric stainless steel on the electrochemical workstation (PGSTAT302N). The EIS frequency range is from 10 MHz to 100 Hz. The data were collected with the temperature ranging from 30 to 80 °C and recorded every 10 °C. The calculation of the ionic conductivity was through Eq. (S1):

$\sigma=\frac{L}{R_{b}\times S}$ (S1)

σ, L, and S represent the ionic conductivity, the electrolyte thickness and the effective contact area between the SS and electrolytes, respectively.

Linear sweep voltammetry (LSV) was used to evaluate the electrochemical stability of the in-situ prepared SPEs. The Li|SPEs|SS cells were tested at a scan rate of 0.1 mV s^−1^ over the range of 0-7 V at 60 °C. The cells were polarized at 10 mV (ΔV) to determine the currents of the initial (*I*_0_) and the steady state (*I*_S_) until the current was steady (10000 s). Interfacial resistances were obtained before (*R*_0_) and after (*R*_S_) polarization. Lithium symmetric cells based on the in-situ formed GPEs were assembled to test the plating-stripping behavior and the lithium-ion transference number (*t*_Li+_) at 60 °C, and the *t*_Li+_ was calculated by Eq. (S2):

$$t_{\mathrm{Li}^{+}}=\frac{I_{S}(\Delta V-I_{0}R_{0})}{I_{0}(\Delta V-I_{S}R_{S})} (S2)$$

The LiFePO_4_ cathode was prepared using a mixed slurry of LFP, Super P and PVDF in NMP with the weight ratio of 8:1:1. The NCM622 cathodes were prepared with a similar method using *N*-methyl-2-pyrrolidone (NMP) as the solvent and PVDF as the binder. The obtained cathodes were dried at 80 ^o^C for 12 h and cut into 12 mm diameter and the average mass ratio of the active material is 1~2.5 mg cm^-2^. All the above-mentioned CR2032 coin cells are in-situ prepared using the TF4030 cellulose separators (diameter 15 mm, thickness 30 μm) as the separator, and the Li plates (diameter 15 mm, thickness 1 mm) were used as the anodes. The long-term cycling and rate performances of the in-situ prepared Li/Polymer@COF/LFP cells were tested with the voltage range of 2.5~4.2 V at 60 °C.

The plating-stripping process of the *in-situ* prepared Li/Polymer@COF/Li cell was tested with a current density of 0.1 mA cm^−2^ at 60 °C. The galvanostatic charge-discharge performances of the symmetric Li cells were tested in the charge-discharge rate of 0.1~ 0.5 mA cm^−2^.

**S4 Finite element analysis simulation**

Specific simulation process and methods refer to literature [S3]. In this simplified model performed via COMSOL Multiphysics 6.2. The Nernst-Planck equation is used to describe the mass transfer (mass conservation) and charge conservation of active Li+ in the electrolyte. The ion mass transfer is considered, including the diffusion caused by the concentration gradient and the electromigration caused by electricity. A square region was established as study area and the current density was set to 1 mA cm^−2^. First, based on the morphology of the Ex-situ or in-situ Polymer electrolyte distribution in the AFM and SEM, construct geometric models corresponding to Ex-situ P(4CL-TMC)@COF and In-situ P(4CL-TMC)@COF, respectively, with the randomness of particle positions reflecting the COF distribution characteristics. Subsequently, the upper and lower boundaries of the model are defined as the electrodes. Also, and the concentration of Li^+^ in the model is calculated by Eq. (S3):

$i_{l}=i_{0}\{\exp\left( \frac{\alpha F\eta}{RT} \right)-c_{0}\exp\left( \frac{\beta F\eta}{RT} \right)\}$(S3)

Here, $i_{l}$represents the local current density, $i_{0}$denotes the exchange current density, 𝜂 is the reaction over potential, 𝛼 and 𝛽 are the charge transfer coefficients, and$c_{0}$refers to the initial concentration of the electrolyte. Subsequently, the model calculates mass transfer processes, including electro migration and diffusion, as defined by Eq. (S4):

$J=-D\nabla c - zuFc\nabla\varphi$ (S4)

Finally, by employing the Faraday constant, the concentration and current density are coupled as outlined in Equation, thus facilitating the solution for the transient ion concentration distribution by Eq. (S5):

$i = zFJ$ (S5)

**S5 MSD Computational Methods**

All the calculations are performed in the framework of the molecular dynamics, as implemented in the LAMMPS Molecular Dynamics Simulator [S4]. The calculation results were visually analyzed using OVITO software [S5]. The Universal Force Field (UFF) was used to descrtbe the interaction force between atoms [S6]. The topological information of the initial model was obtained using the Packmol and Moltemplate programs [S7, S8]. In the UFF (Universal Force Field), the interactions between atoms can be described by Eq. (S6) ~ (S11). Prior to conducting the simulation, the first step is to perform energy minimization on the model, ensuring that the system starts in a relatively stable initial energy state. Subsequently, the model is placed at a temperature of 300 K and undergoes sufficient relaxation within the NPT ensemble. This process allows the model to reach a state of thermodynamic equilibrium, eliminating any potential unreasonable stresses and structural distortions within the model, thus laying the foundation for the accuracy and reliability of subsequent simulations. During the simulation, the radial distribution function (RDF) and number density distribution (NDD) of each molecule were investigated under the conditions of 300 K and the NVT ensemble.

Bond: (S6)

Angle: (S7)

Improper: (S8)

Van der Waals: (S9)

Electrostatic: (S10)

Total Energy: (S11)

**S6 DFT Computational Methods**

The corrected charges simulations of these molecules were conducted by a Materials Studio software, reversion 2023 within a DFT theory. Following geometry optimization and ESP charge process were operated in DMol3 package. And a pure density functional method M06-L, which is one of the most popular formulations in metageneralized gradient approximation (m-GGA) method was adapt in these simulations. The calculation of desolvation energy use the Eq. (S12):

$E_{Desolvation Energy}=E_{total}-(E_{{Li}^{+}}+E_{other})$ (S12)

Here, $E_{total}$ represent the total energy of the systems after geometry optimization, $E_{{Li}^{+}}$represent the energy of the Li^+^ geometry optimization, and the $E_{other}$ is the energy of the repeat unit of the polymer or part structure of COF. Denoted, in order to reduce the computational complexity, all of the structure of COF and polymer has been reasonably simplified according to the similar structure from literature.


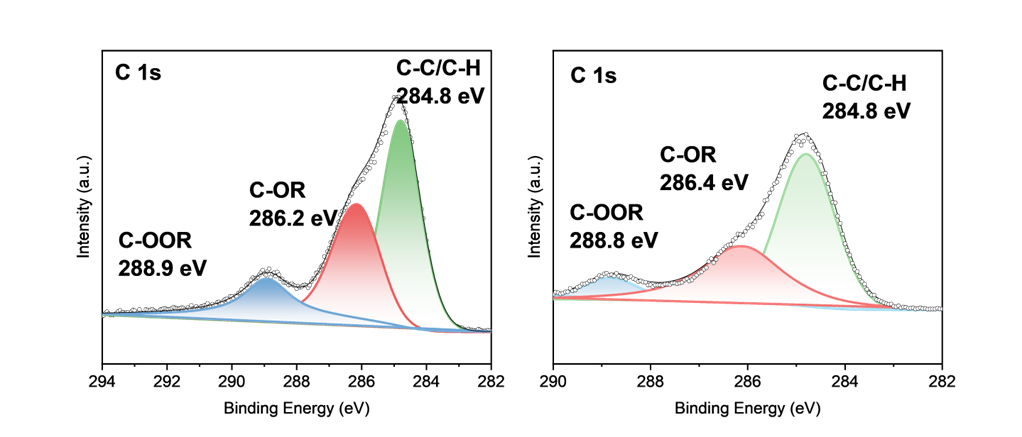


**Fig. S2** XPS (C 1s) spectra of TpPa-COOH (left) and TpPa-COOLi (right)

**
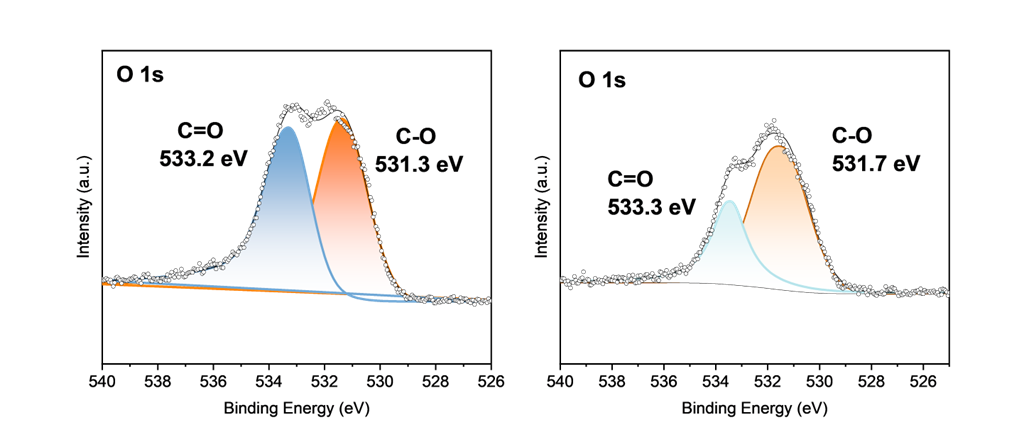
**

**Fig. S3** XPS (O 1s) spectra of TpPa-COOH (left) and TpPa-COOLi (right)


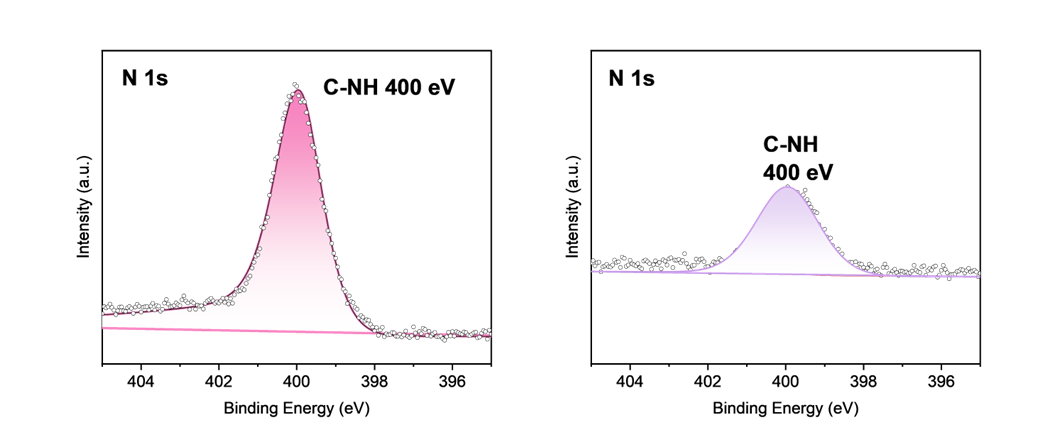


**Fig. S4** XPS (N 1s) spectra of TpPa-COOH (left) and TpPa-COOLi (right)


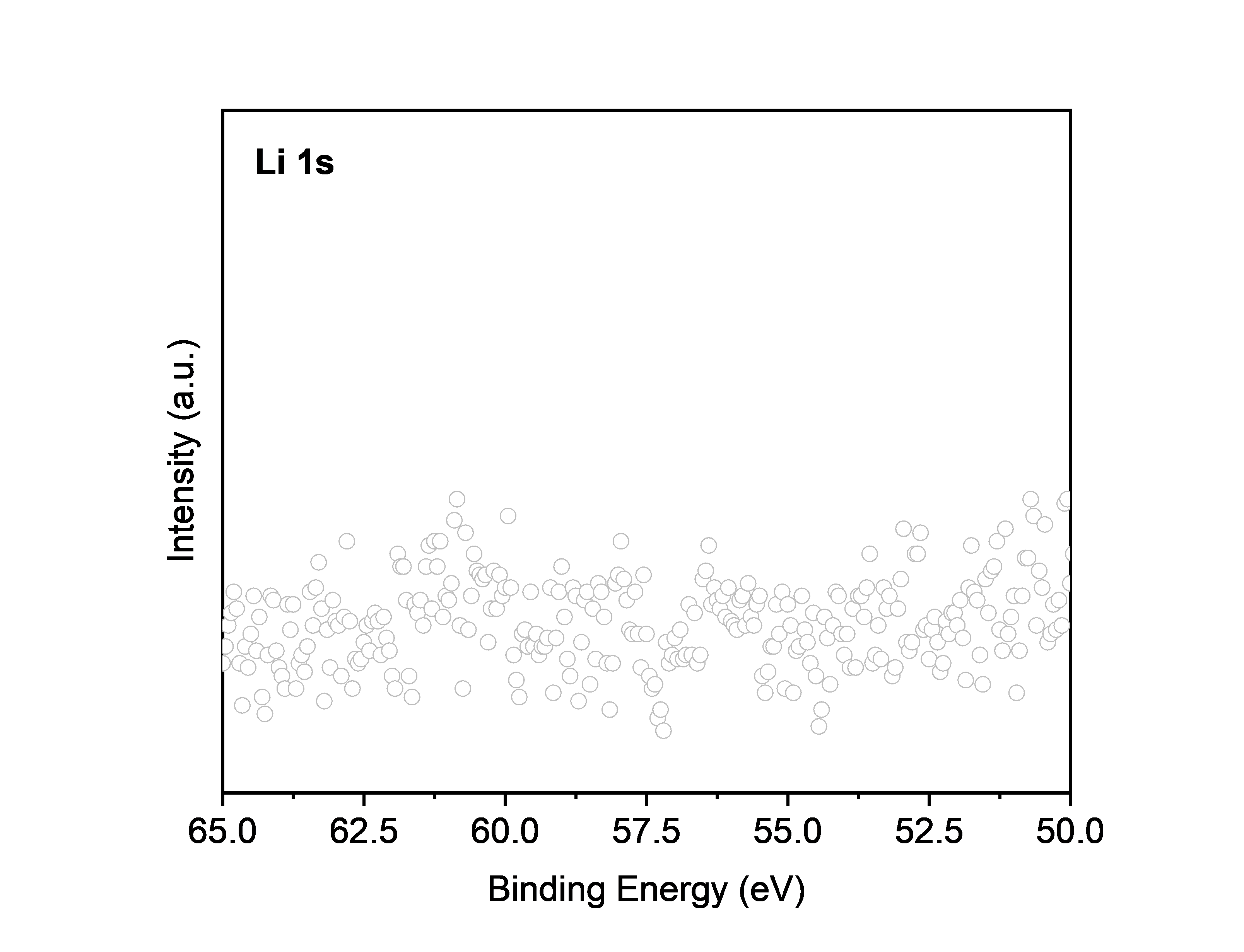


**Fig. S5** XPS (Li 1s) spectra of TpPa-COOH


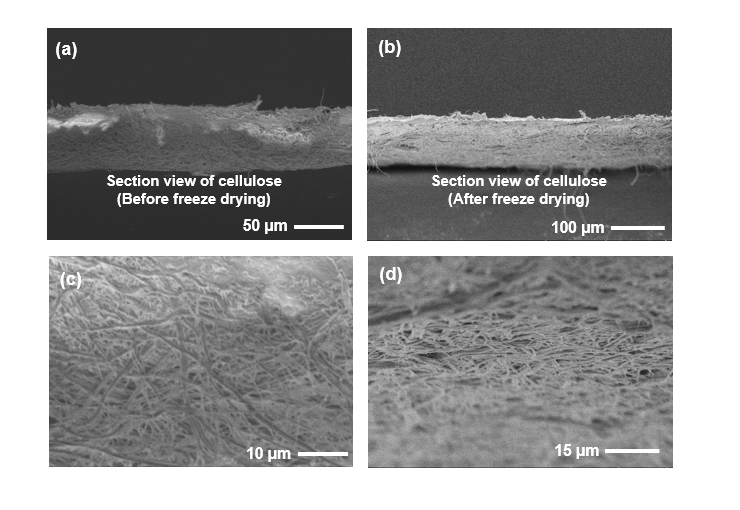


**Fig. S6** **a**) The SEM section view image of cellulose separator before freeze-drying treatment. **b**) The SEM section view image of cellulose separator after freeze-drying treatment. **c**) The local magnification SEM section view image of cellulose separator before freeze-drying treatment. **d**) The local magnification SEM section view image of cellulose separator after freeze-drying treatment


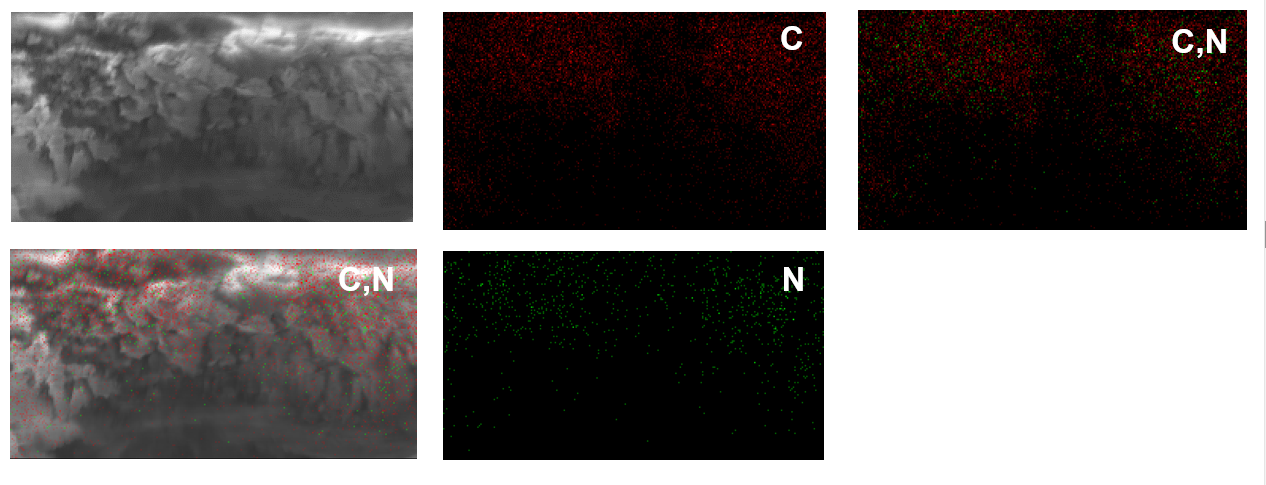


**Fig. S7** EDS characterization of the section view image of cellulose separator loaded COF


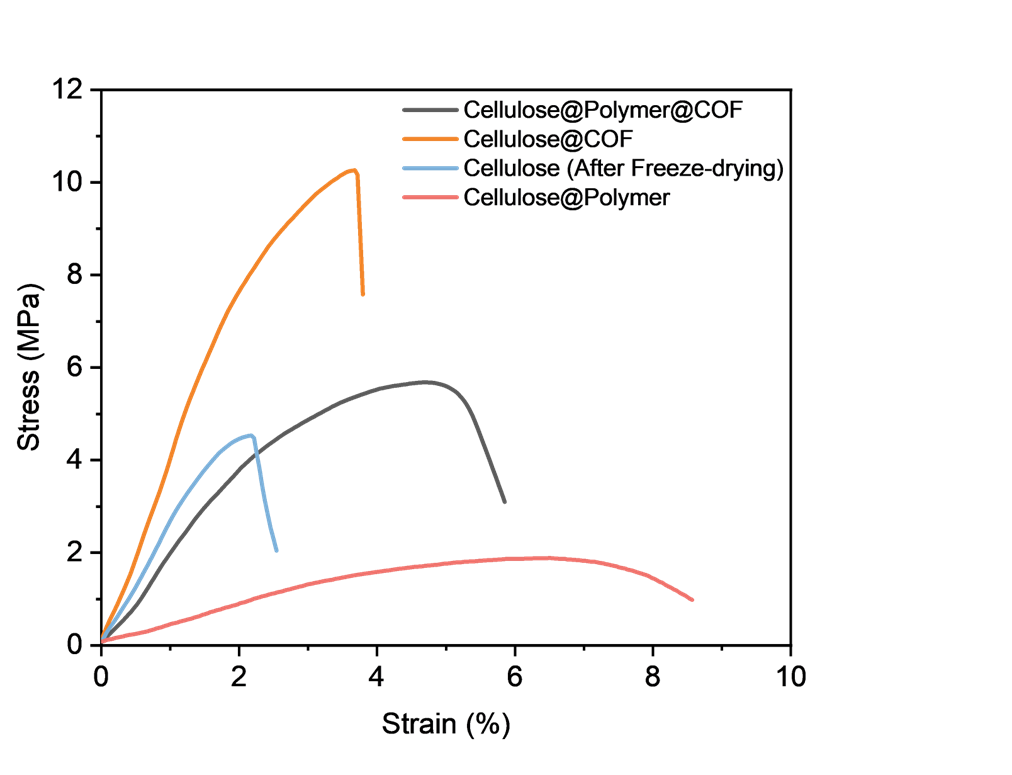


**Fig. S8** Strain-stress curves of Cellulose@Polymer@COF, Cellulose@COF, Cellulose, Cellulose@Polymer


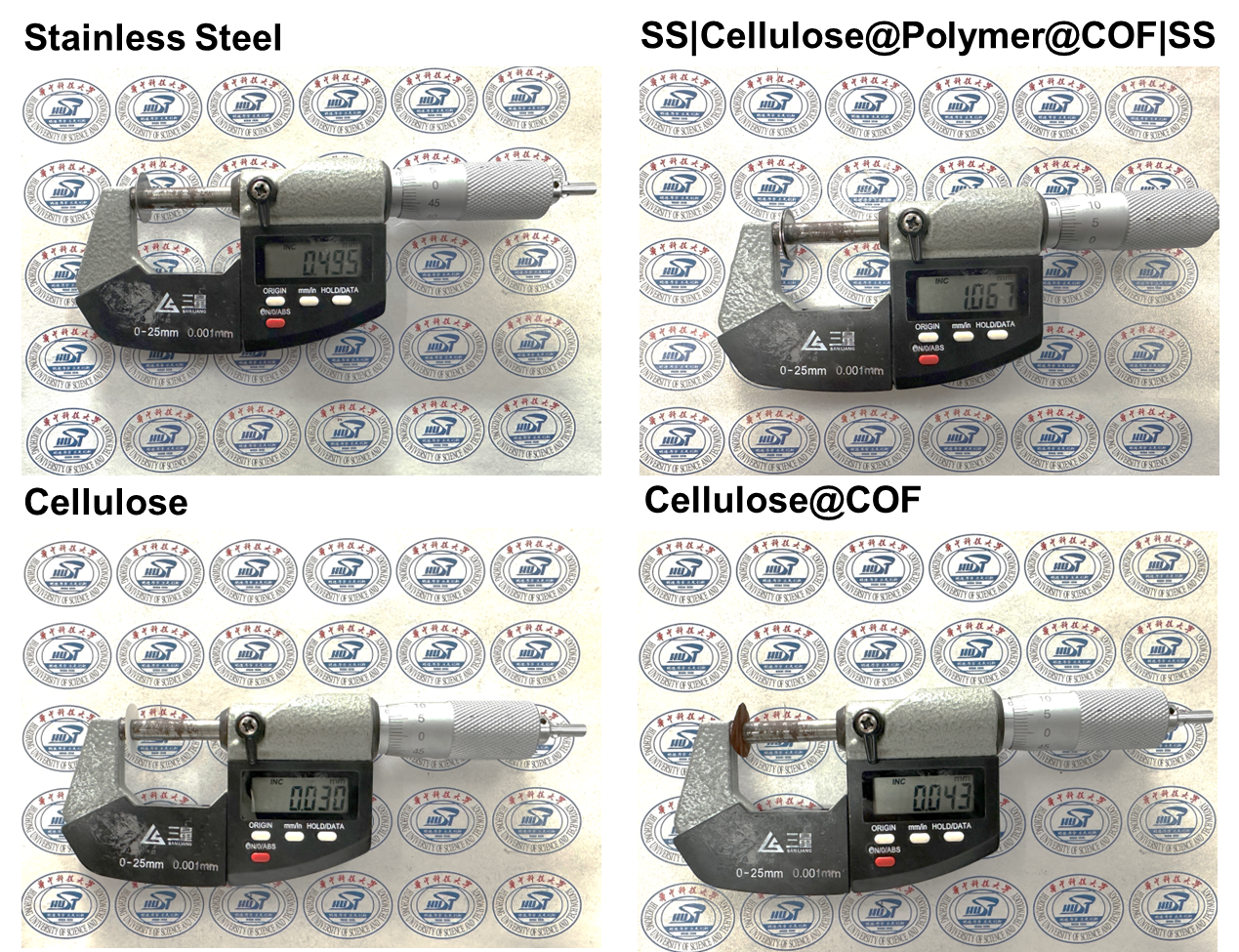


**Fig. S9** Thickness of Cellulose@Polymer@COF, Cellulose, Cellulose@COF

**Table S1** Thickness of Original Cellulose, Cellulose@COF, Cellulose@Polymer@COF

| **Items** | **Thickness (μm)** |
| --- | --- |
| Original Cellulose | 30 |
| Cellulose@COF | 43 |
| Cellulose@Polymer@COF | 77 |


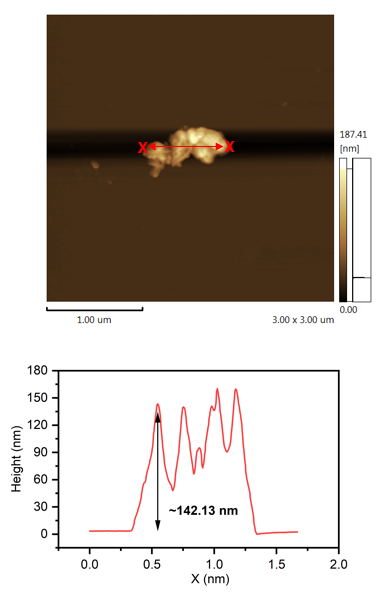


**Fig. S10** AFM image with roughness of COF (TpPa-COOLi)

**
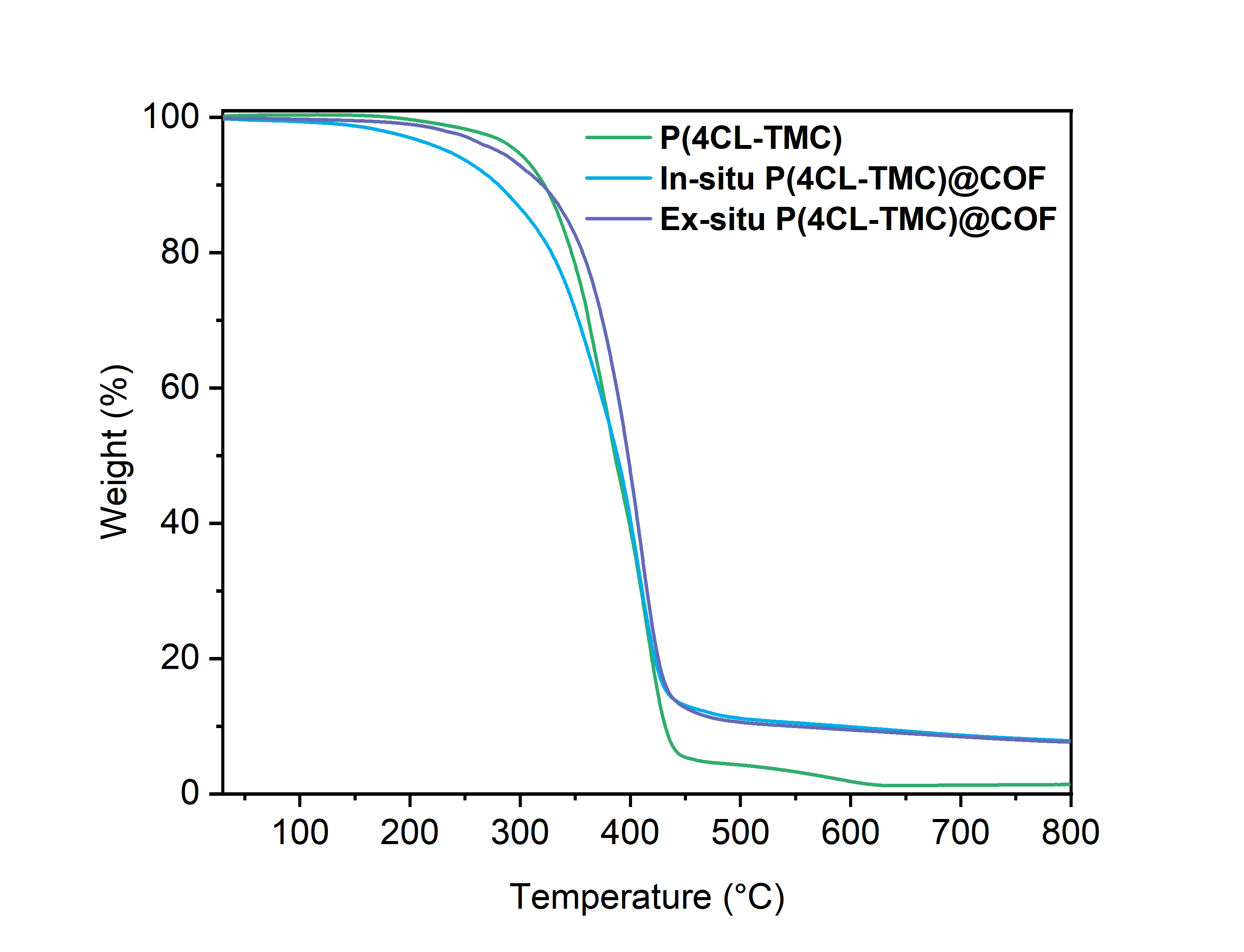
**

**Fig. S11** The TGA curves of the P(4CL-TMC), In-situ P(4CL-TMC)@COF and Ex-situ P(4CL-TMC)@COF

**
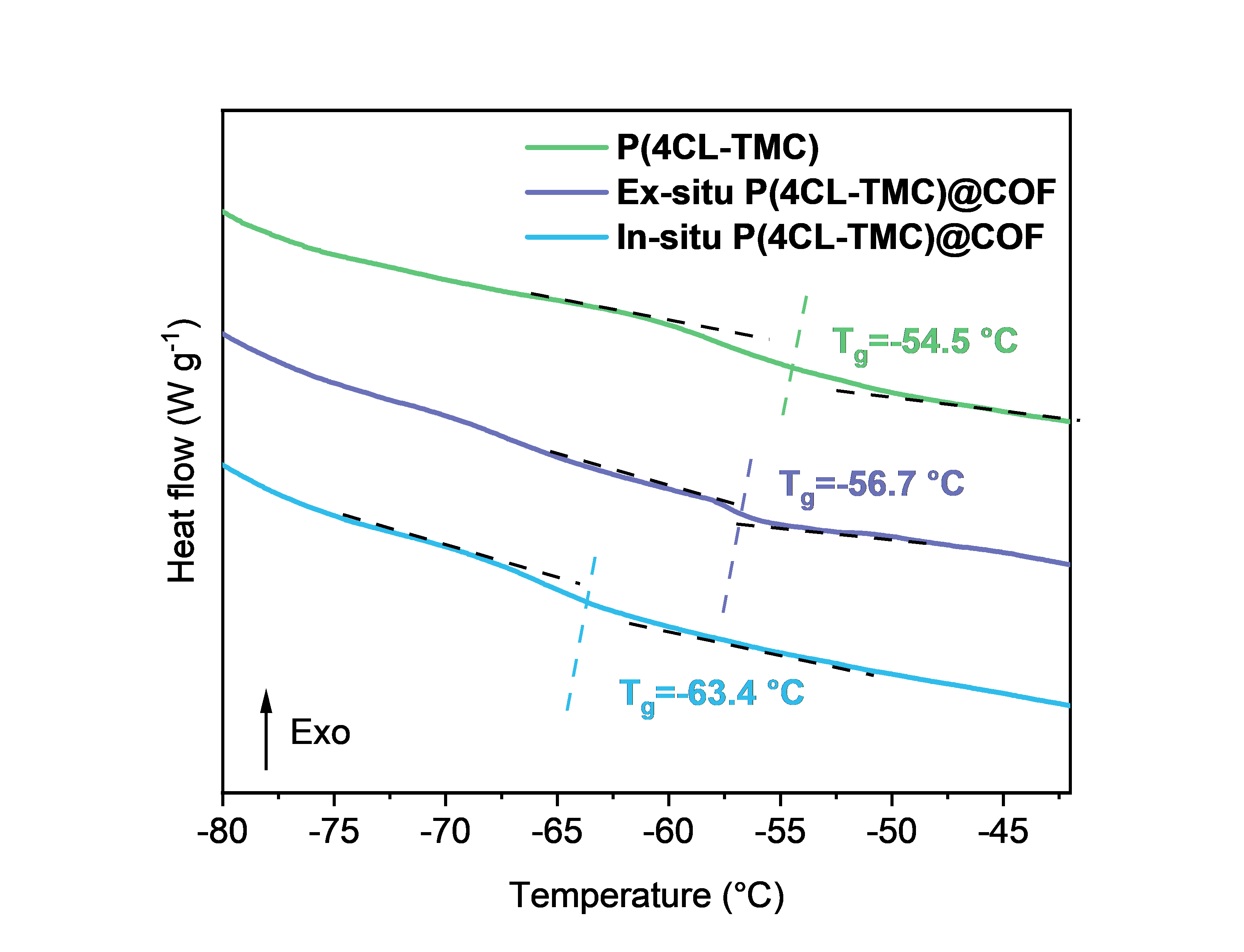
**

**Fig. S12** DSC curves of in-situ P(4CL-TMC)@COF, ex-situ P(4CL-TMC)@COF, and P(4CL-TMC)

**
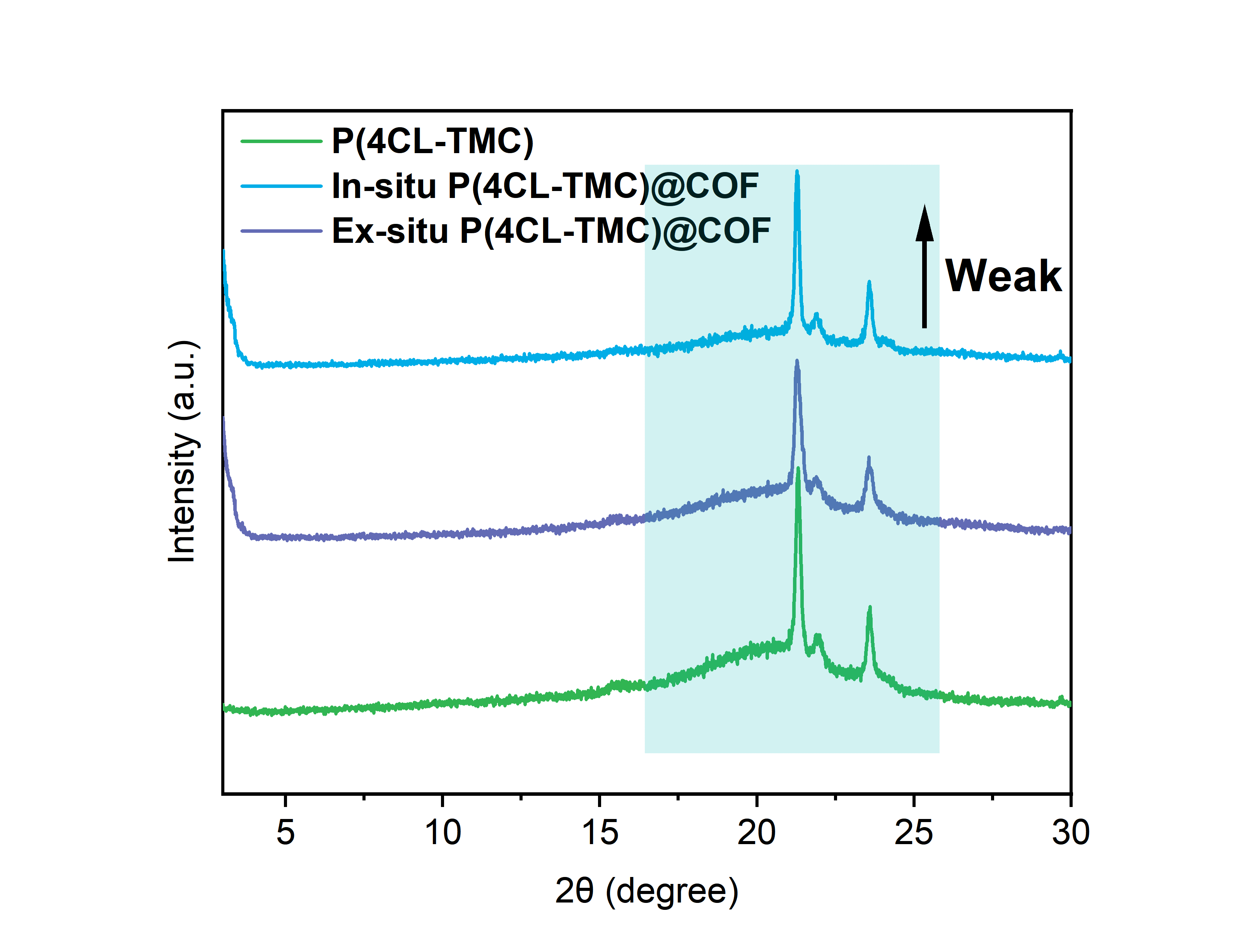
**

**Fig. S13** The XRD characterization of the P(4CL-TMC), In-situ P(4CL-TMC)@COF and Ex-situ P(4CL-TMC)@COF

**
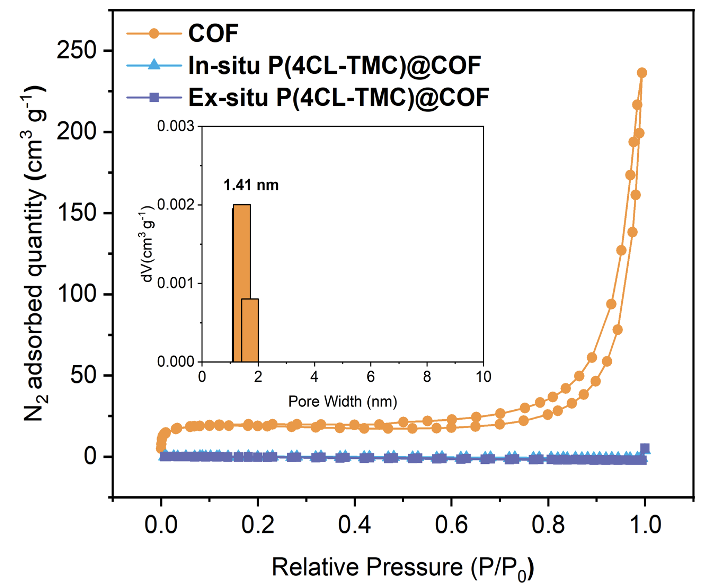
**

**Fig. S14** Nitrogen gas sorption isotherms measured at 77 K for COF (TpPa-COOLi), in-situ P(4CL-TMC)@COF, and ex-situ P(4CL-TMC)@COF

**
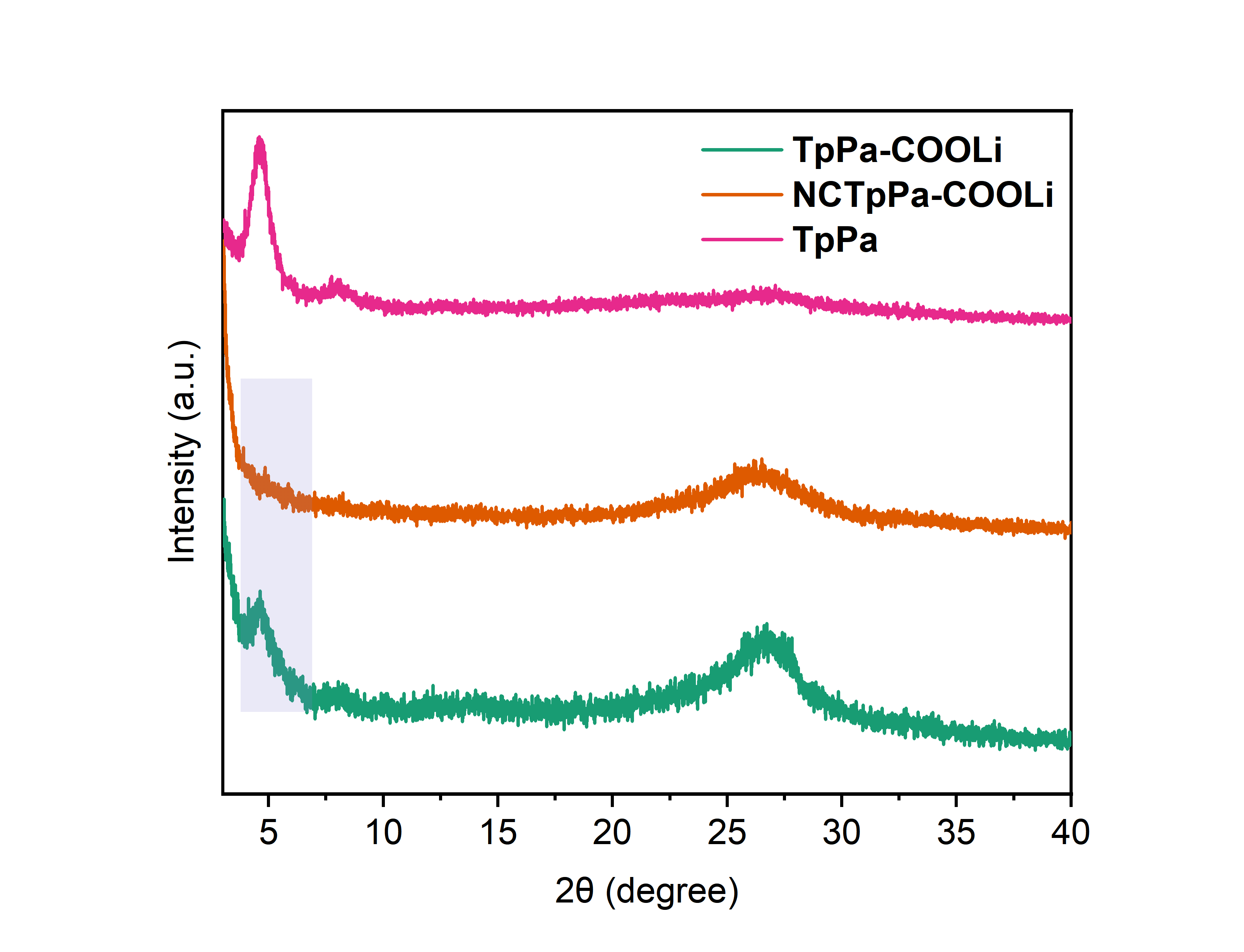
**

**Fig. S15** Powder X-ray diffraction (PXRD) of TpPa-COOLi, NCTpPa-COOLi and TpPa

**
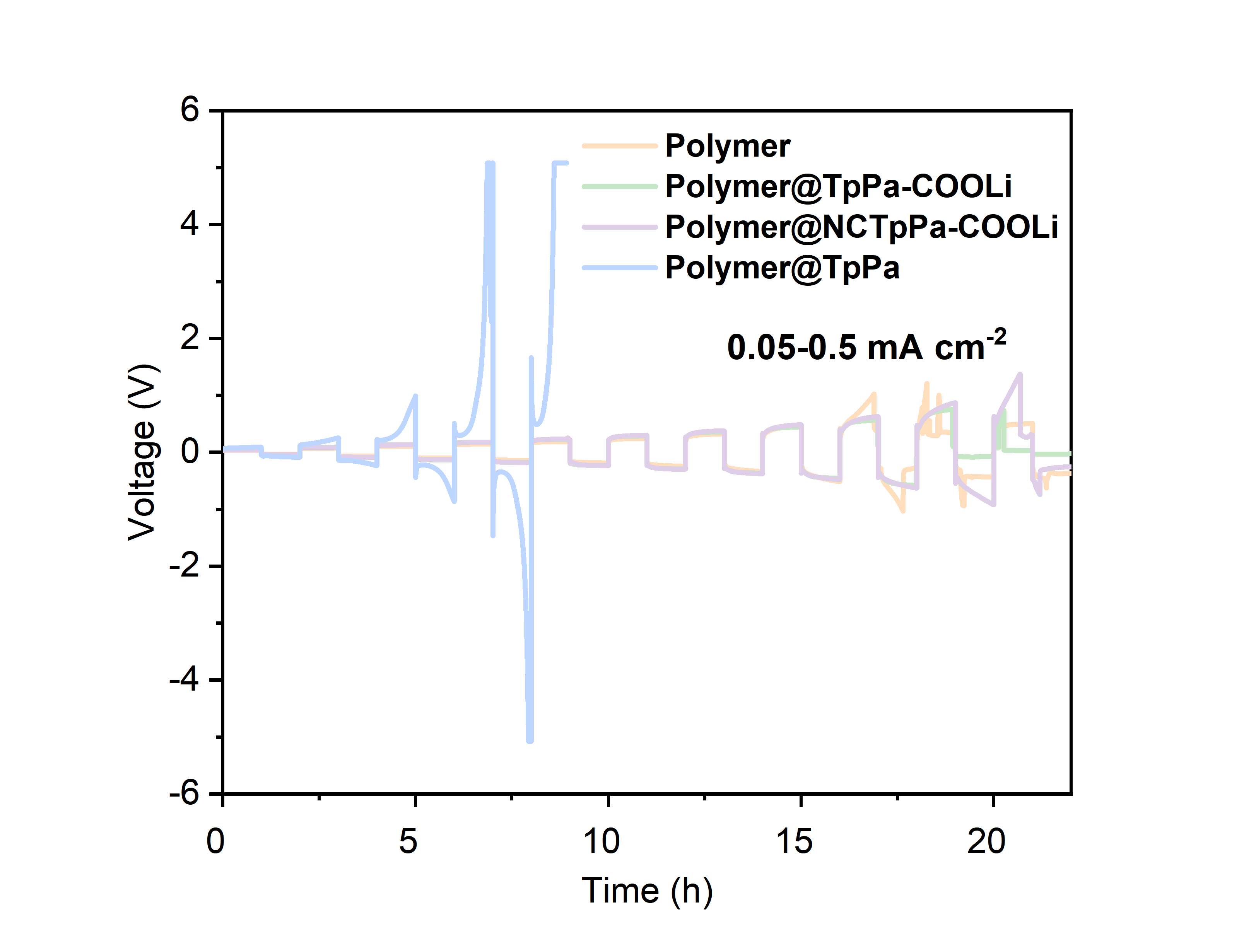
**

**Fig. S16** The curves of critical current density of Polymer, Polymer@TpPa-COOLi, Polymer@NCTpPa-COOLi and Polymer@TpPa

**
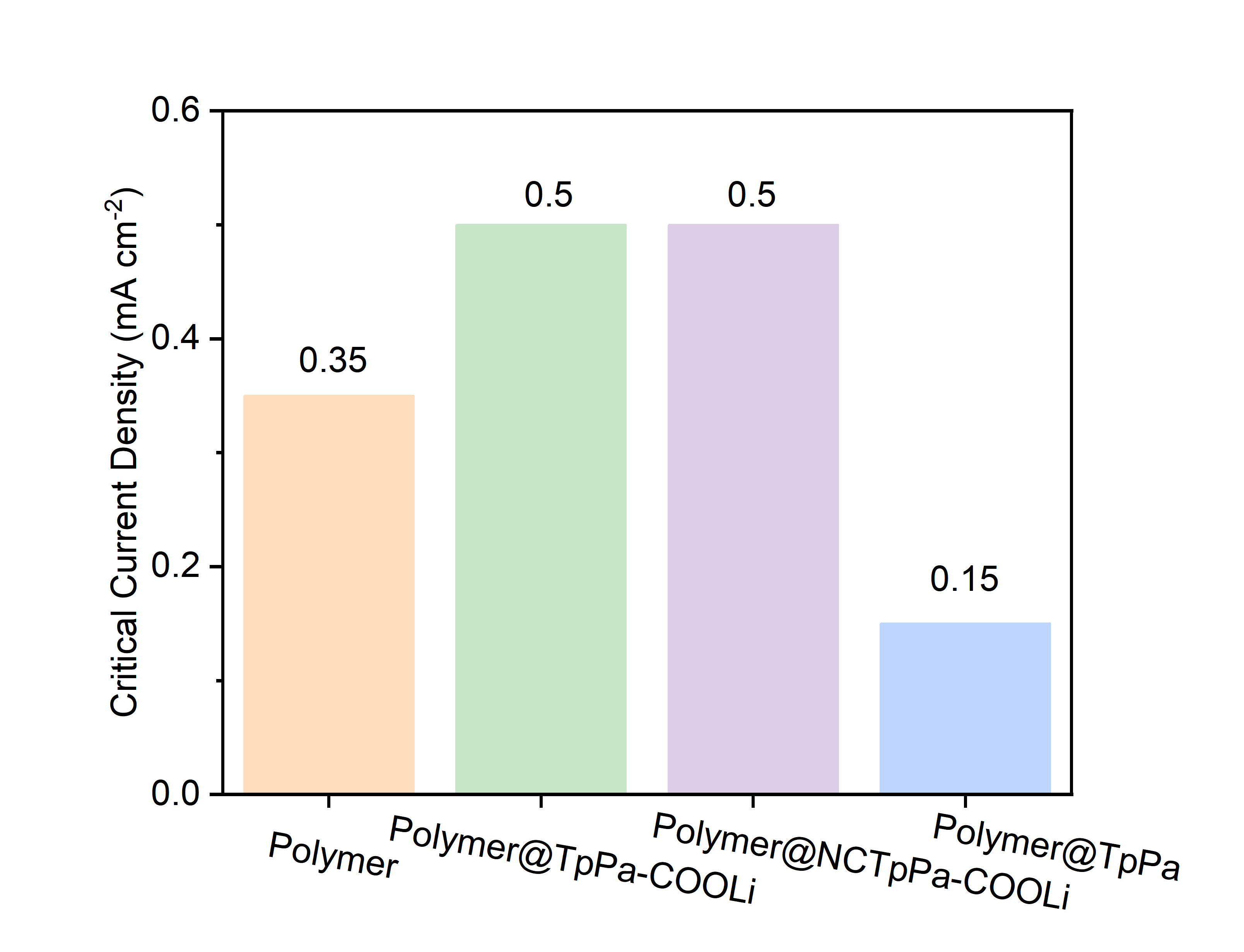
**

**Fig. S17** The bar chart of critical current density of Polymer, Polymer@TpPa-COOLi, Polymer@NCTpPa-COOLi and Polymer@TpPa

**
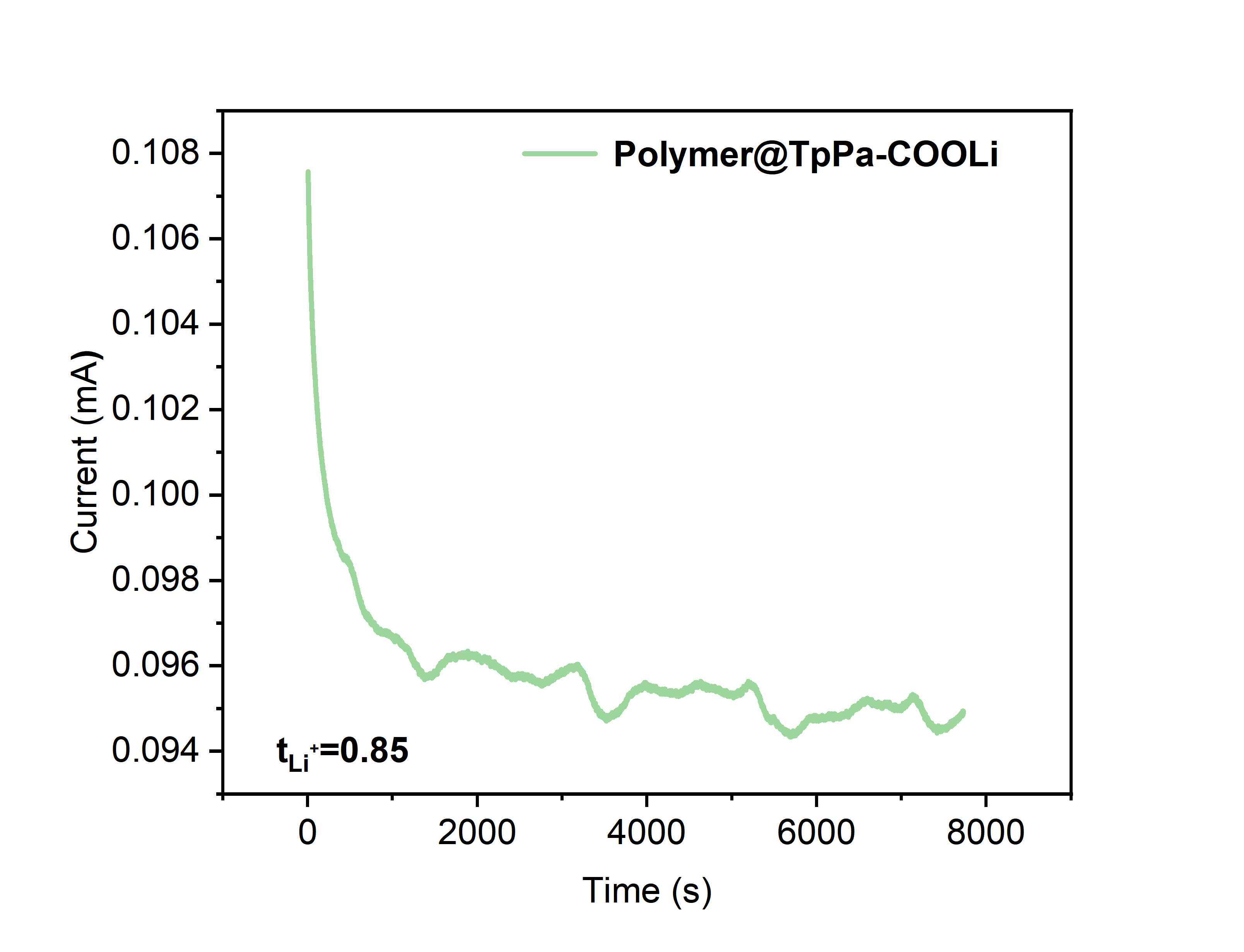
**

**Fig. S18** The CA curves of Li/Polymer@TpPa-COOLi/Li symmetric batteries at 60 °C

**
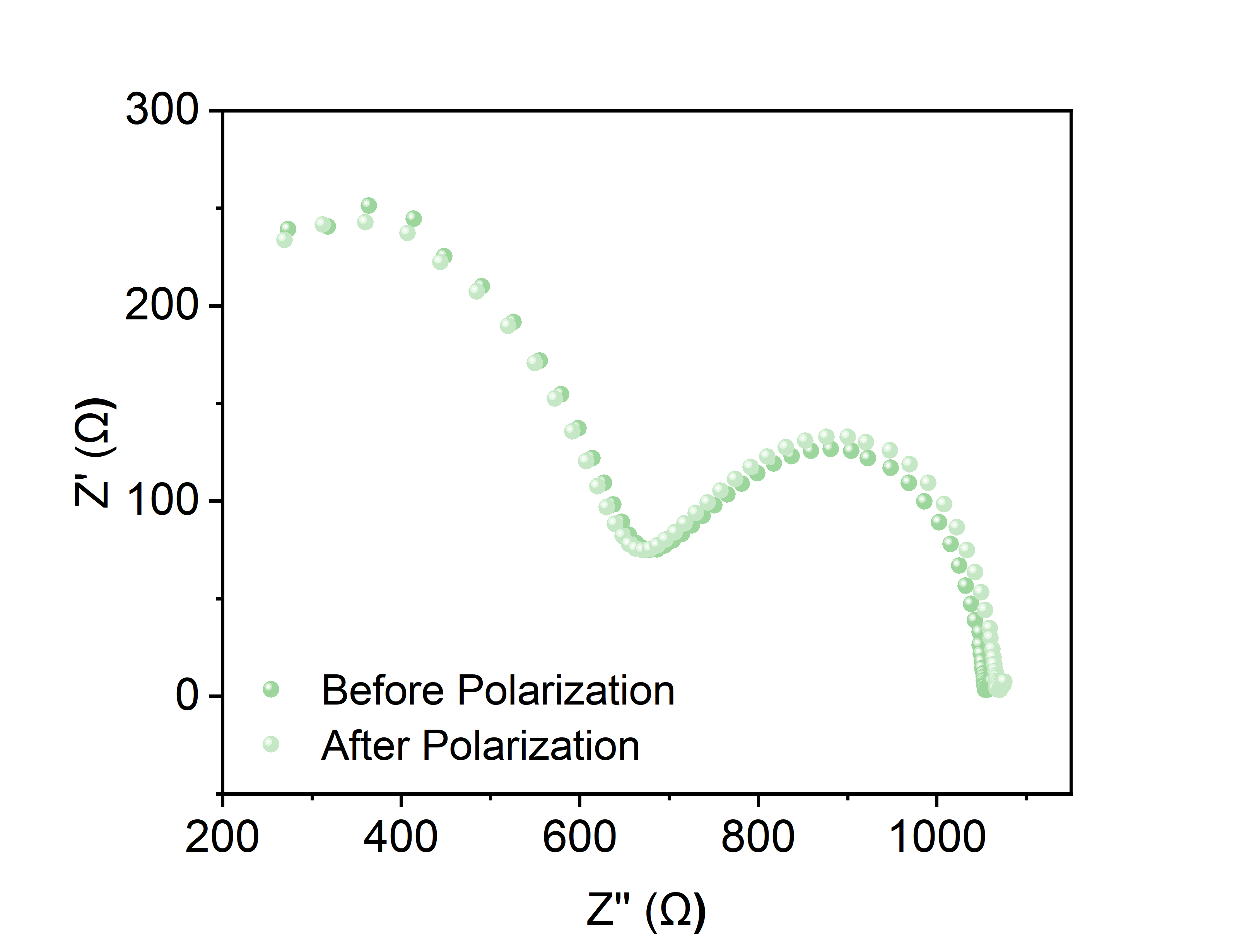
**

**Fig. S19** Impedance diagram before and after polarization of Li/Polymer@TpPa-COOLi/Li symmetric batteries at 60 °C

**
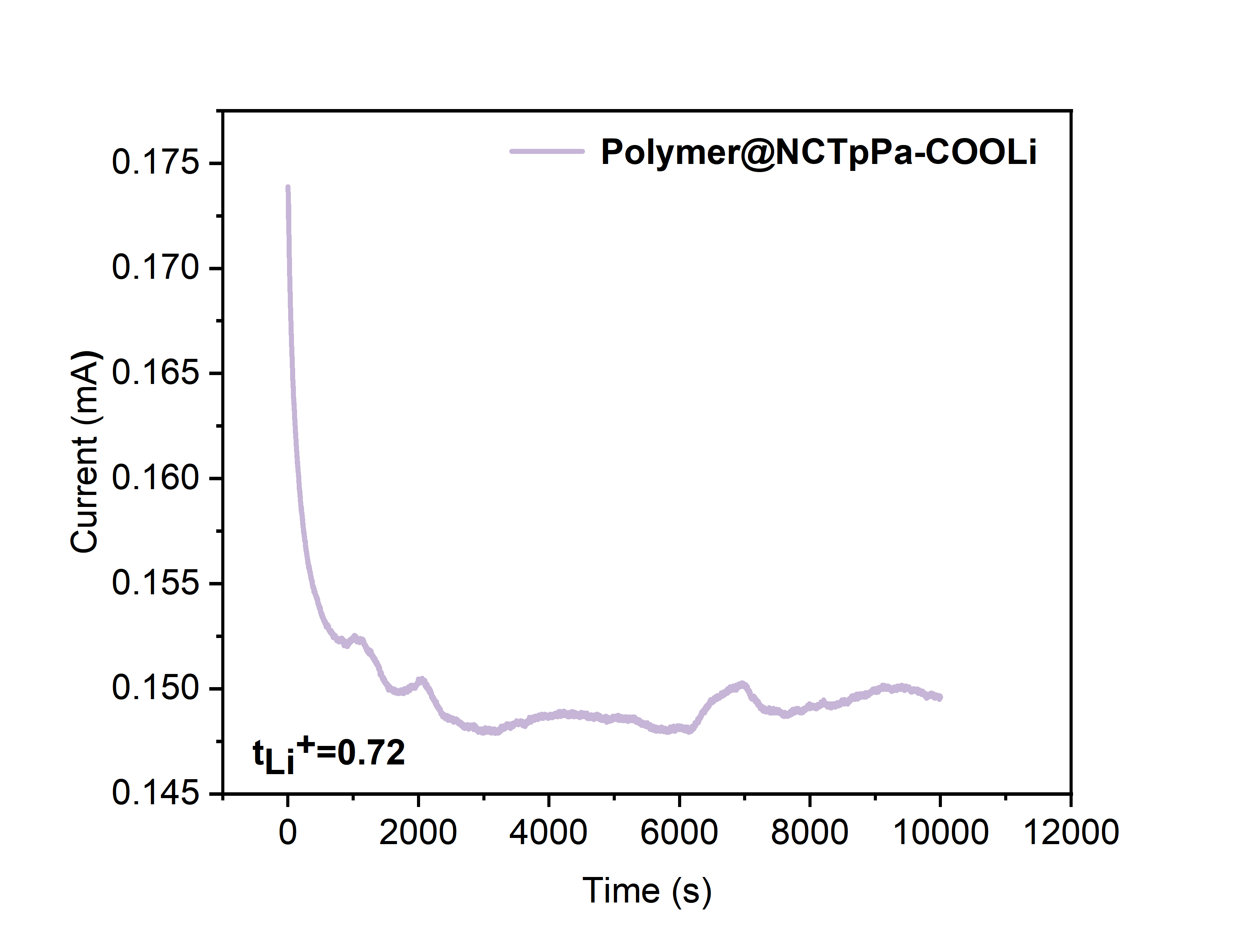
**

**Fig. S20** The CA curves of Li/Polymer@NCTpPa/Li symmetric batteries at 60 °C

**
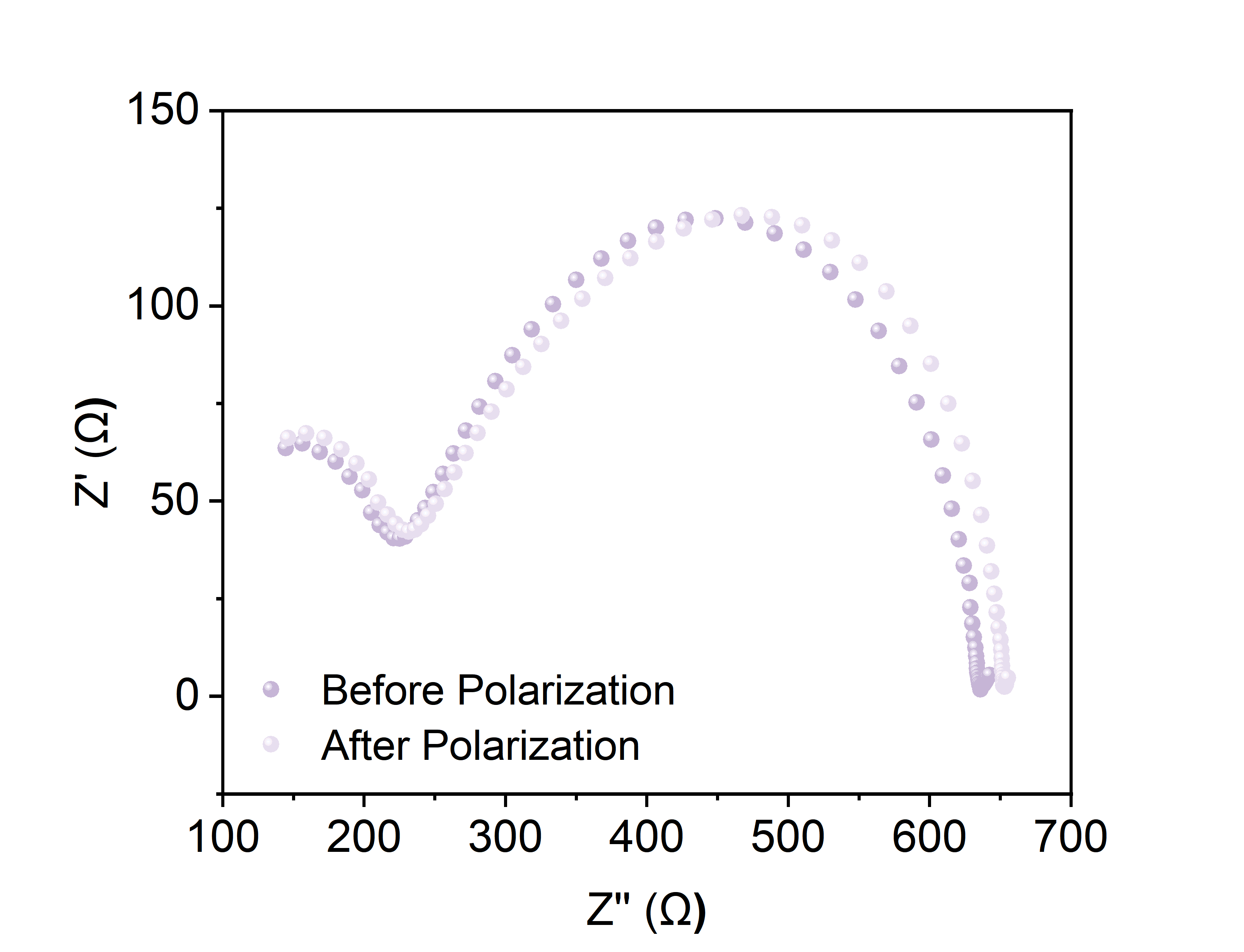
**

**Fig. S21** Impedance diagram before and after polarization of Li/Polymer@NCTpPa-COOLi/Li symmetric batteries at 60 °C

**
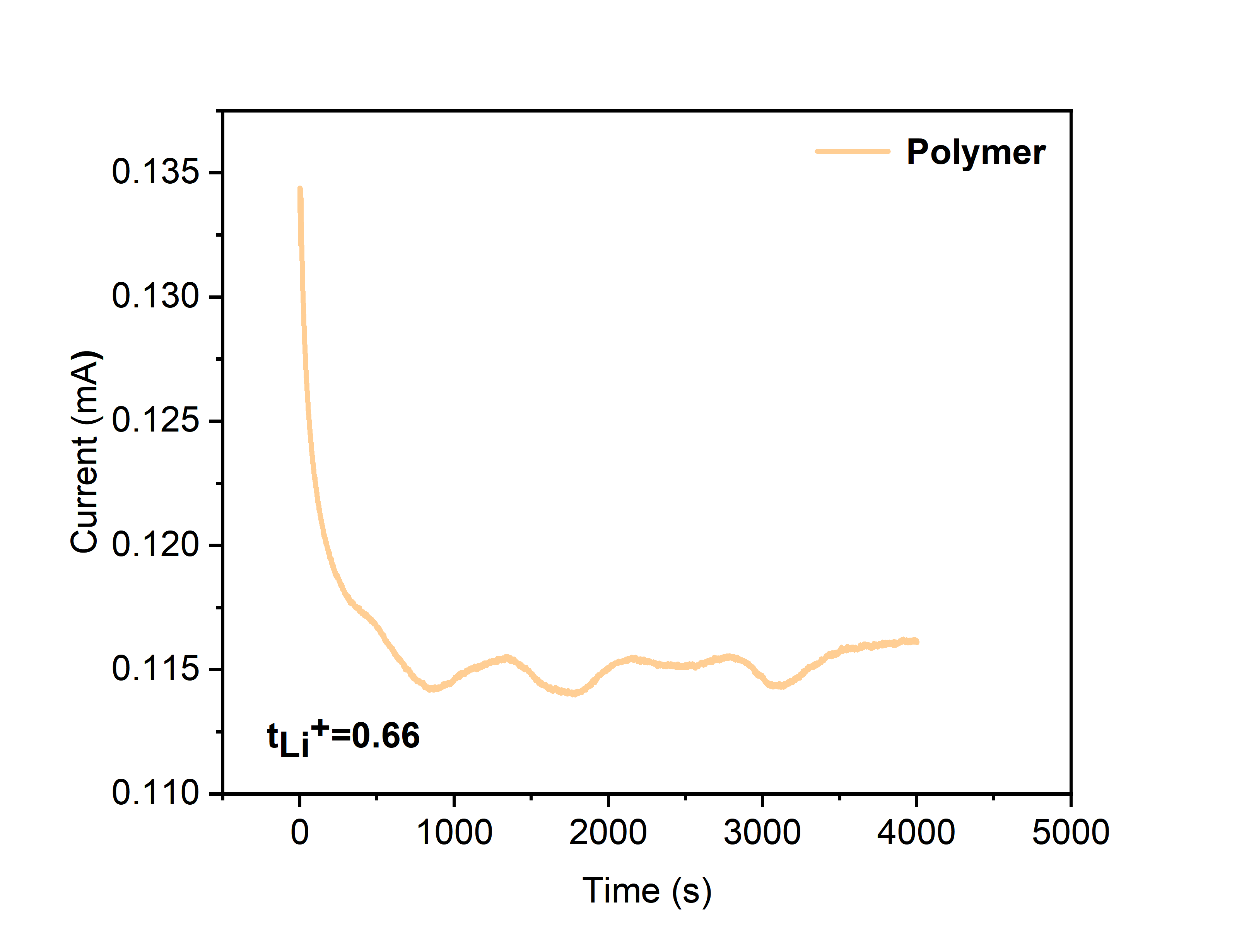
**

**Fig. S22** The CA curves of Li/Polymer/Li symmetric batteries at 60 °C

**
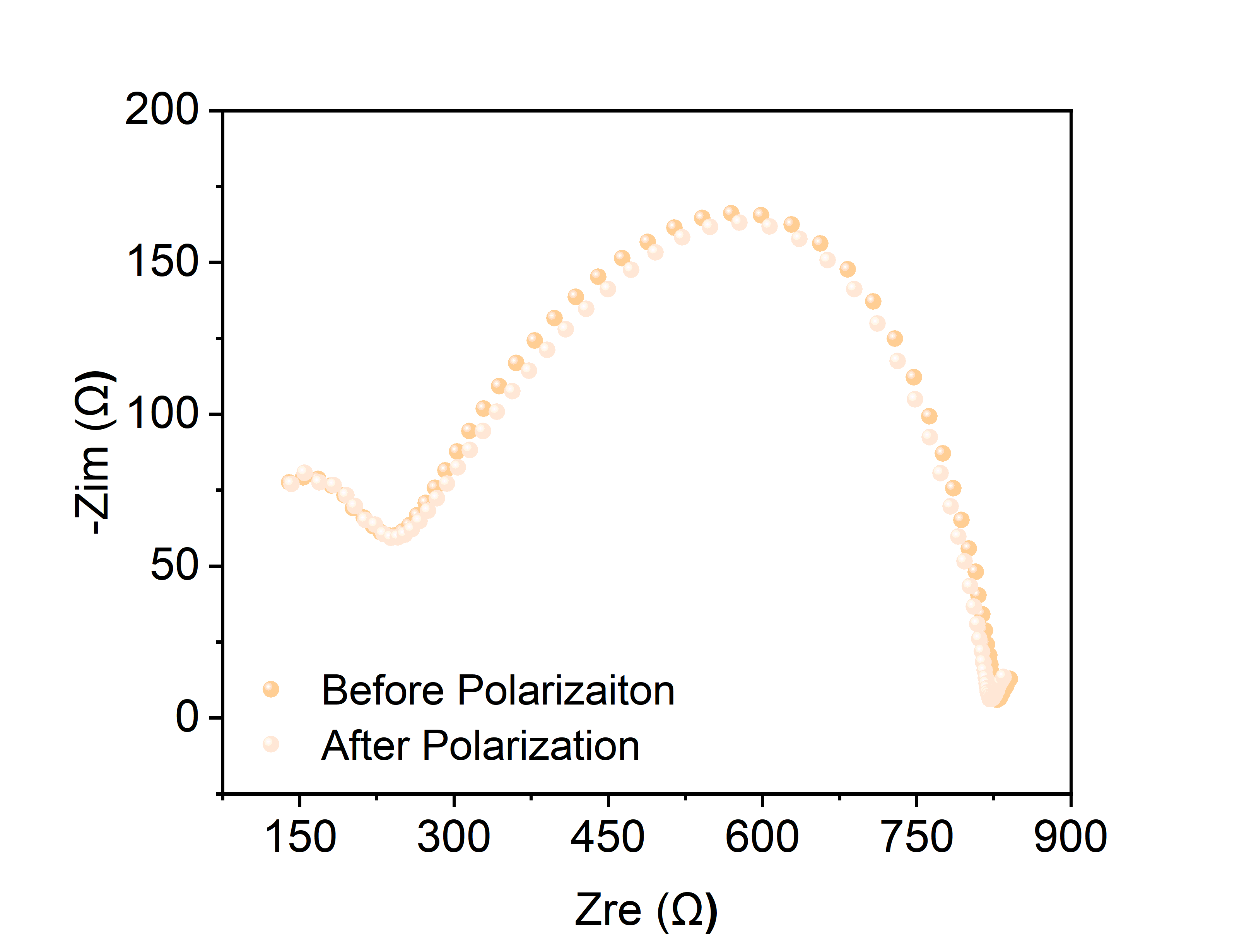
**

**Fig. S23** Impedance diagram before and after polarization of Li/Polymer/Li symmetric batteries at 60 °C

**
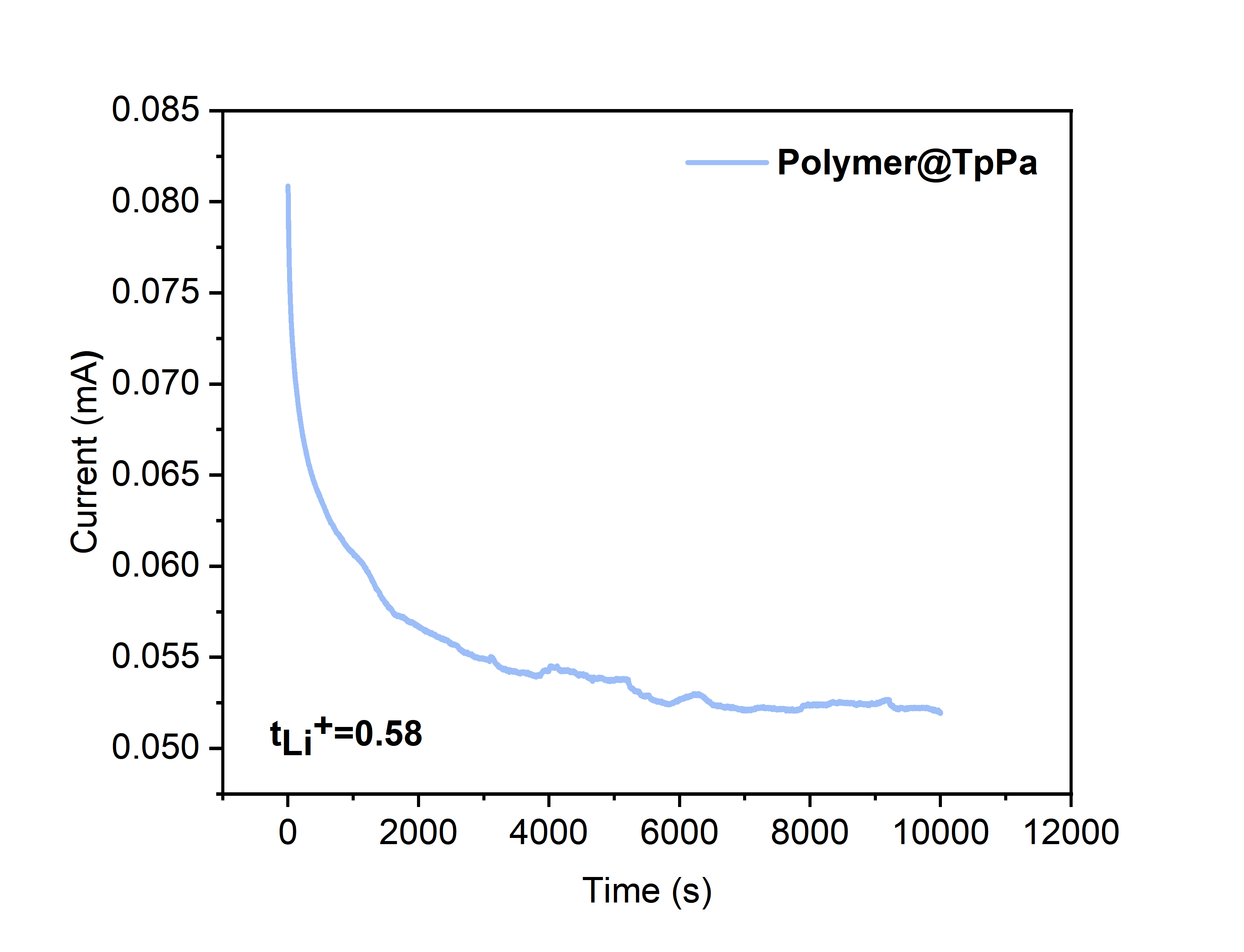
**

**Fig. S24** The CA curves of Li/Polymer@TpPa/Li symmetric batteries at 60 °C

**
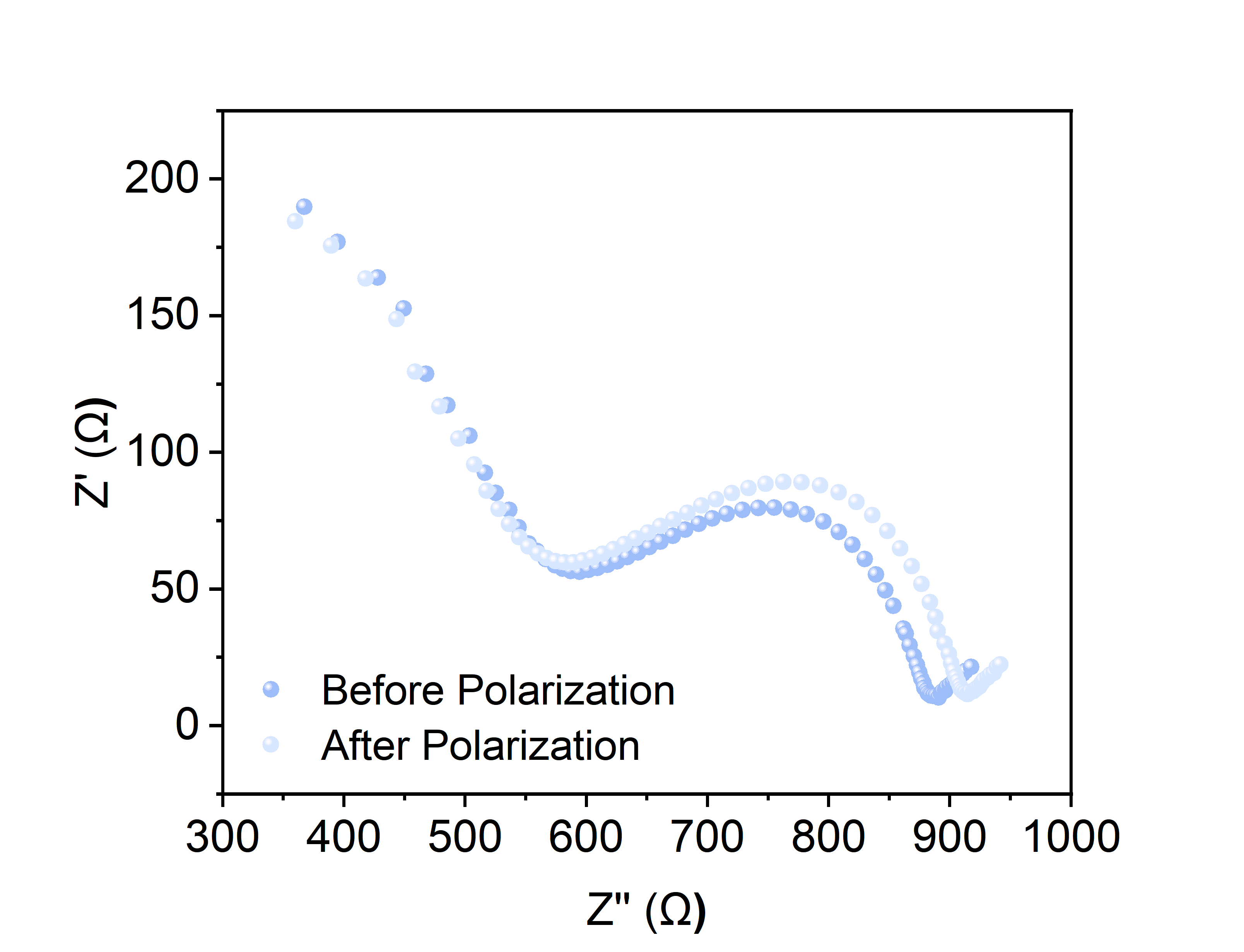
**

**Fig. S25** Impedance diagram before and after polarization of Li/Polymer@TpPa/Li symmetric batteries at 60 °C

**
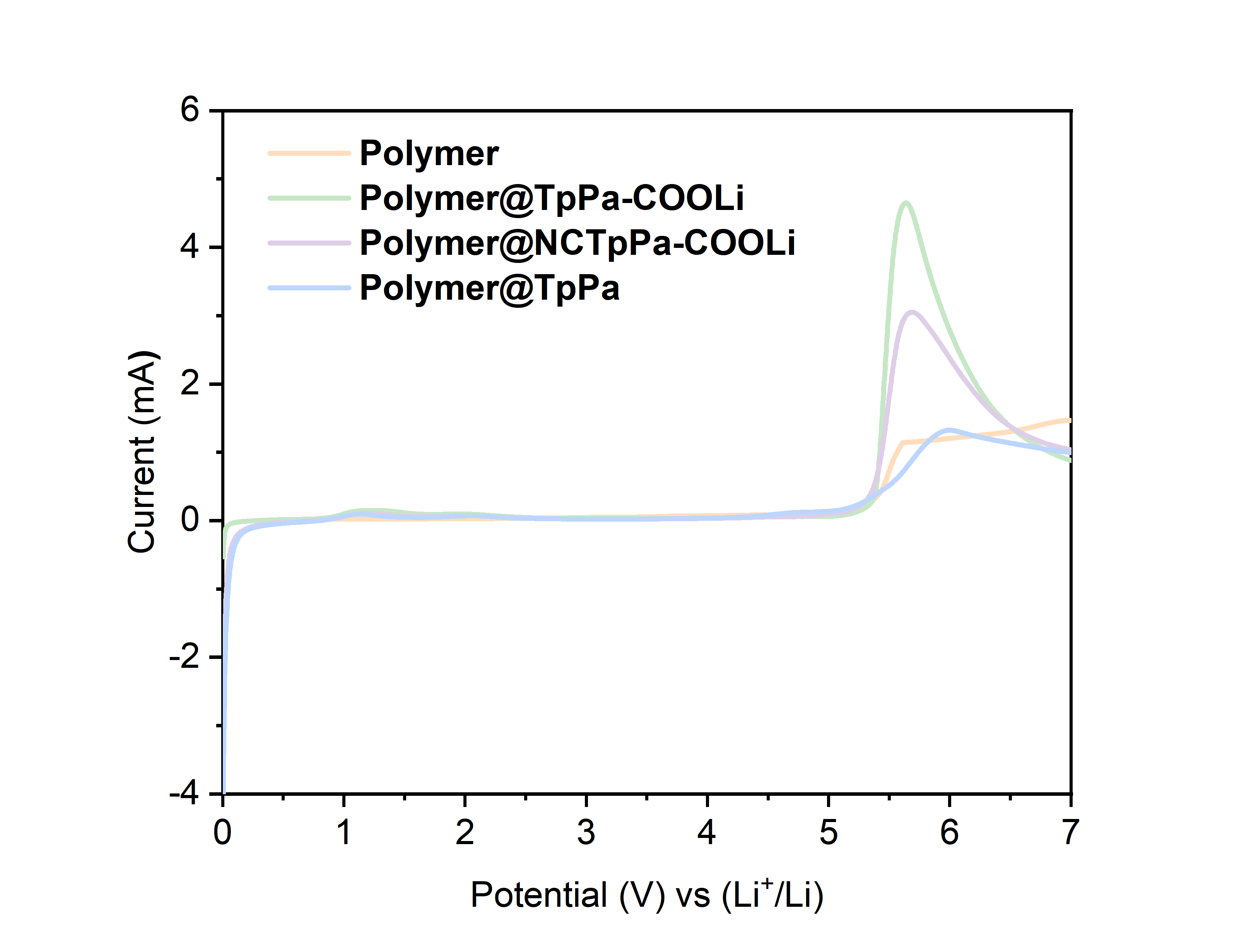
**

**Fig. S26** LSV curves of polymer, polymer@TpPa-COOLi, polymer@NCTpPa-COOLi, and polymer@TpPa

**
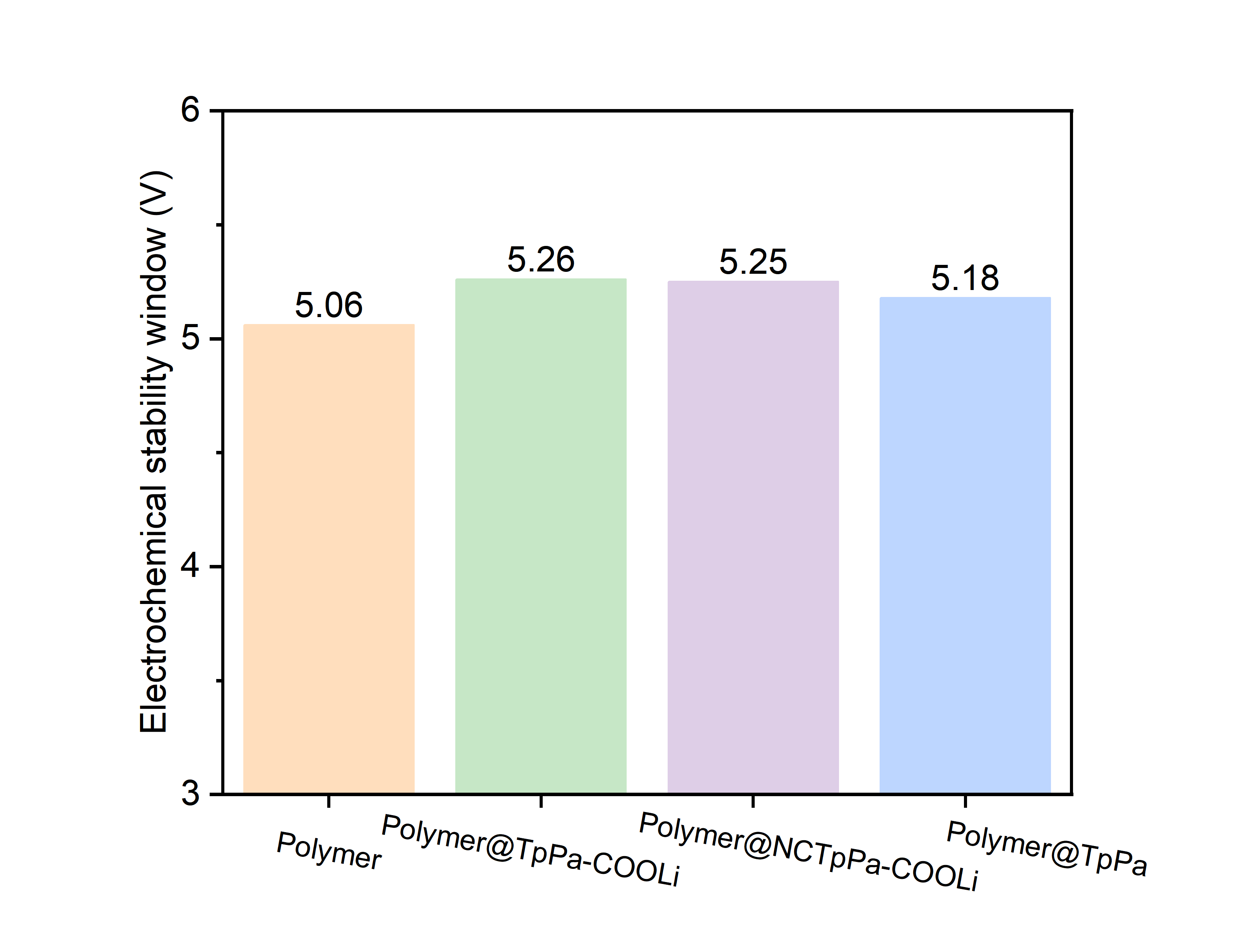
**

**Fig. S27** The bar chart of electrochemical stability window of Polymer, Polymer@TpPa-COOLi, Polymer@NCTpPa-COOLi and Polymer@TpPa

**
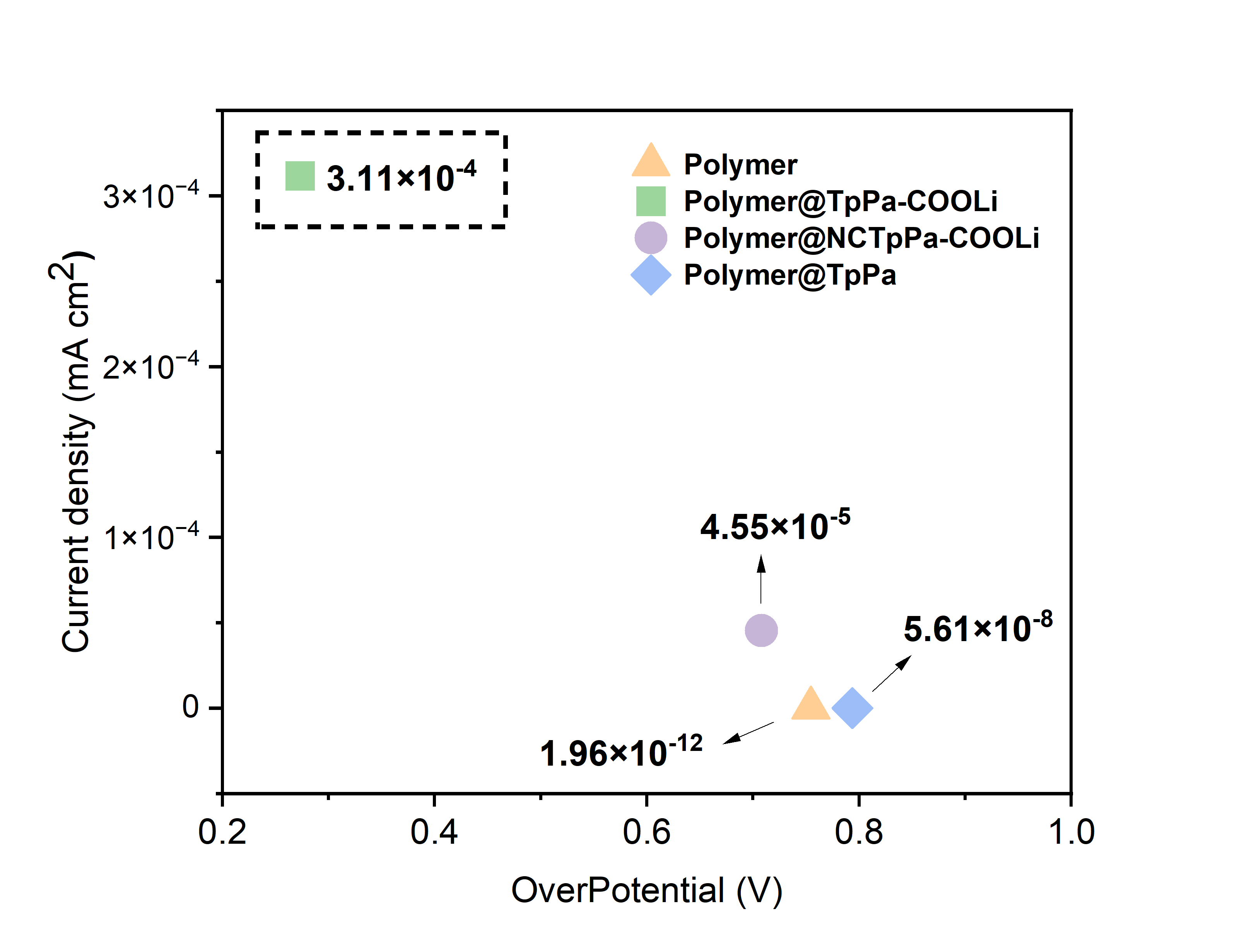
**

**Fig. S28** Plot of exchange current density versus overpotential of Polymer, Polymer@TpPa-COOLi, Polymer@NCTpPa-COOLi and Polymer@TpP

**
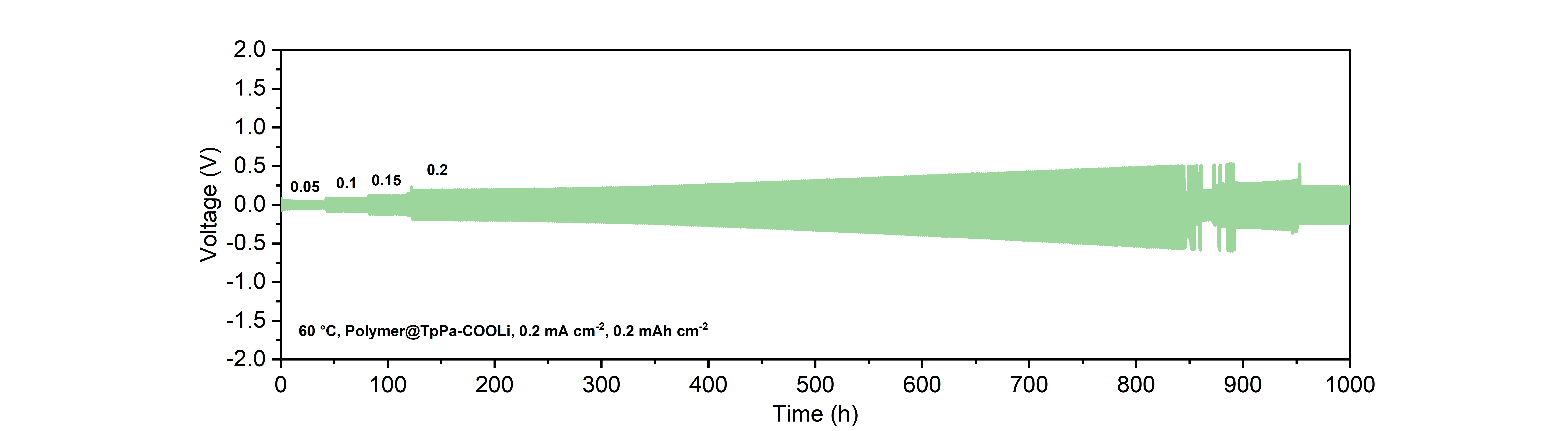
**

**Fig. S29** Voltage profiles of Li/Polymer@TpPa-COOLi/Li symmetric cell with the current density of 0.05-0.2 mA cm^-2^ at 60 °C

**
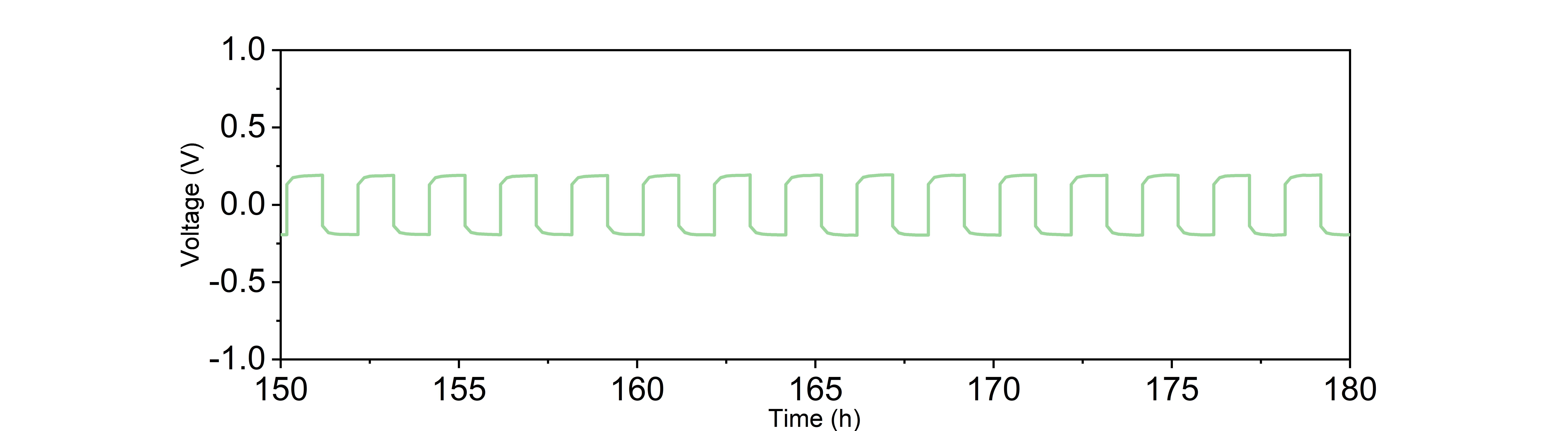
**

**Fig. S30** Voltage profiles of Li/Polymer@TpPa-COOLi/Li symmetric cell with the current density of 0.05-0.2 mA cm^-2^ at 60 °C. Enlarged voltage profiles at 150-180 h


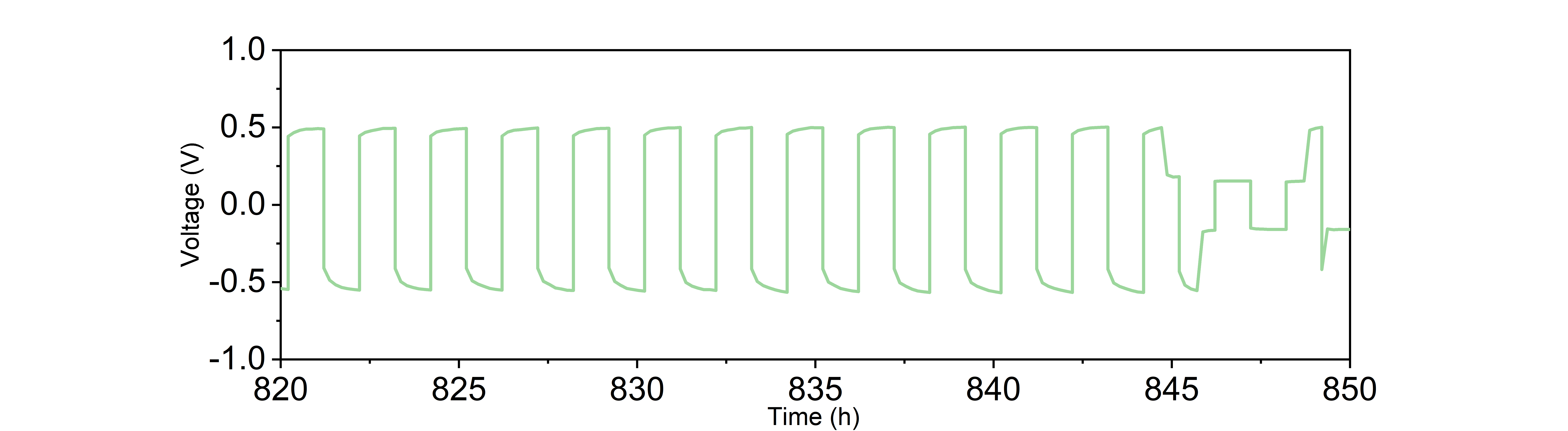
**Fig. S31** Voltage profiles of Li/Polymer@TpPa-COOLi/Li symmetric cell with the current density of 0.05-0.2 mA cm^-2^ at 60 °C. Enlarged voltage profiles at 820-850 h


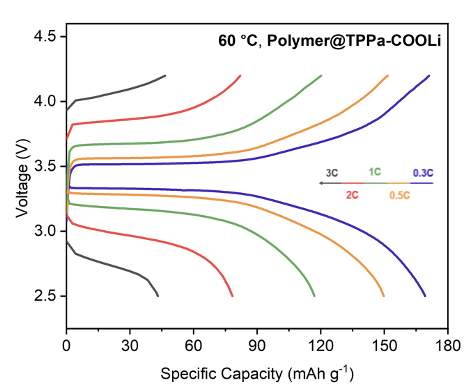


**Fig. S32** Charge–discharge curves of Polymer@TpPa-COOLi half-cells at varied current density (0.1C, 0.3C, 0.5C, 1C, 2C, 3C) under 60 °C

**
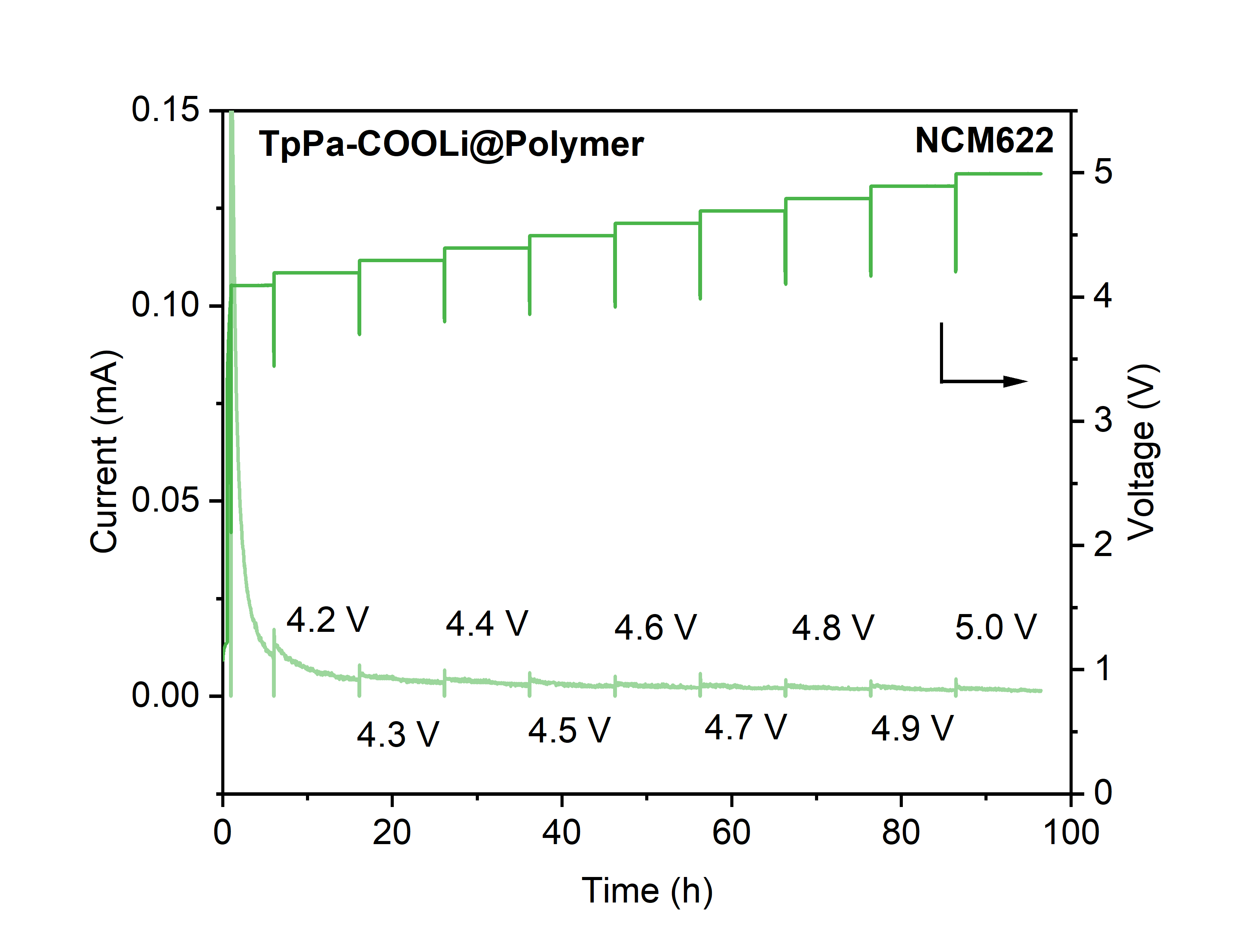
**

**Fig. S33** Electrochemical floating test curve of Li/Polymer@TpPa-COOLi/NCM622 half-cells

**Supplementary References**

1. G. Zhao, Z. Mei, L. Duan, Q. An, Y. Yang et al., COF-based single Li+ solid electrolyte accelerates the ion diffusion and restrains dendrite growth in quasi-solid-state organic batteries. Carbon Energy **5**(2), e248 (2023). <https://doi.org/10.1002/cey2.248>
2. A. Mei, H. Guo, W. Zhang, Y. Liu, W. Chen, Regulating water adsorption sites of keto-enamine COF by base exfoliation and deprotonation for enhanced humidity response. Small **20**(43), 2403521 (2024). <https://doi.org/10.1002/smll.202403521>
3. Y. Feng, Y. Fan, L. Zhao, J. Yu, Y. Liao et al., Enhancing microdomain consistency in polymer electrolytes towards sustainable lithium batteries. Angew. Chem. Int. Ed **64**(5), e202417105 (2025). <https://doi.org/10.1002/anie.202417105>
4. A.P. Thompson, H.M. Aktulga, R. Berger, D.S. Bolintineanu, W.M. Brown et al., LAMMPS - a flexible simulation tool for particle-based materials modeling at the atomic, meso, and continuum scales. Comput. Phys. Commun. **271**, 108171 (2022). <https://doi.org/10.1016/j.cpc.2021.108171>
5. A. Stukowski, Visualization and analysis of atomistic simulation data with OVITO-the Open Visualization Tool. Model. Simul. Mater. Sci. Eng. **18**(1), 015012 (2010). <https://doi.org/10.1088/0965-0393/18/1/015012>
6. A.K. Rappe, C.J. Casewit, K.S. Colwell, W.A. Goddard III, W.M. Skiff, UFF, a full periodic table force field for molecular mechanics and molecular dynamics simulations. J. Am. Chem. Soc. **114**(25), 10024–10035 (1992). <https://doi.org/10.1021/ja00051a040>
7. L. Martínez, R. Andrade, E.G. Birgin, J.M. Martínez, PACKMOL: a package for building initial configurations for molecular dynamics simulations. J. Comput. Chem. **30**(13), 2157–2164 (2009). <https://doi.org/10.1002/jcc.21224>
8. A.I. Jewett, D. Stelter, J. Lambert, S.M. Saladi, O.M. Roscioni et al., Moltemplate: a tool for coarse-grained modeling of complex biological matter and soft condensed matter physics. J. Mol. Biol. **433**(11), 166841 (2021). <https://doi.org/10.1016/j.jmb.2021.166841>
